# Designing Physical Unclonable Functions From Optically Active Materials

**DOI:** 10.1002/adma.202502059

**Published:** 2025-06-09

**Authors:** Maxime Klausen, Junfang Zhang, Molly M. Stevens

**Affiliations:** ^1^ Kavli Institute for Nanoscience Discovery Department of Physiology Anatomy and Genetics Department of Engineering Science University of Oxford Oxford OX1 3QU UK

**Keywords:** anticounterfeiting, nano/micropatterning, optical probes, physical unclonable functions, security devices

## Abstract

Physical unclonable functions (PUFs) are artificial “fingerprints” provided by physical devices to authenticate manufactured goods. Their inherent unclonable nature positions them as one of the most promising tools to tackle global counterfeiting challenges. Leveraging the large parameter space in solution chemistry, chemically generated PUFs can achieve excellent device performance. Particularly, optically active materials have become valuable security inks thanks to their versatile, non‐invasive, and non‐destructive readouts, and PUF devices generated from stochastic nano‐/micro‐patterns of optical inks hold great potential. This review highlights recent advances in the design of optical PUF devices. A range of resonant and non‐resonant optical materials used as security taggants are presented and their incorporation in state‐of‐the‐art PUF devices is examined using non‐deterministic fabrication techniques. By outlining design criteria, challenges, and opportunities, a roadmap is provided for developing next‐generation PUFs using established and emerging optical probes and help advance security and reliability in anticounterfeiting technologies.

## Graphical abstract

Assigning unforgeable “fingerprints” to manufactured goods is a key strategy to fight global counterfeiting. Optical physical unclonable functions (PUFs) are chemically generated random patterns of optically active materials serving as unique authenticators. Here, recent advances in optical PUF devices are presented for anticounterfeiting via an overview of available optical taggants and compatible fabrication techniques.

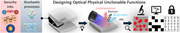

## 1 Introduction

Counterfeiting is a fast‐growing global issue, causing trillion‐dollar economic losses every year. Moreover, counterfeit medicines, certificates, and electronics directly threaten public health and national security. The World Health Organization (WHO) reports that 10% of the global pharmaceutical trade in developing countries is substandard or counterfeited, especially antibiotics and antimalarial agents.^[^ ^1^ ^]^ The consequences of counterfeit medicine on global health are heavy. Indeed, a peer‐reviewed model developed by the University of Edinburgh has estimated that 72 000 to 169 000 children may be dying each year from pneumonia due to substandard and falsified antibiotics.^[^ ^2^ ^]^ Similarly, a model from the London School of Hygiene and Tropical Medicine shows that 116 000 additional deaths from malaria could be caused every year by substandard and falsified antimalarials in sub‐Saharan Africa.^[^ ^2^ ^]^ To eradicate the dramatic consequences of counterfeiting, it is crucial to verify the traceability and authenticity of goods with anticounterfeiting tags. These security tags incorporate unique identifiers and characteristics encoding product authenticity. The core element of such devices is their ability to store, encrypt, and convey security information, which can be achieved by tailored structural design or by using materials exhibiting specific optical properties. Such encryption features make the device difficult to replicate, thereby protecting against unauthorized duplication or forgery. Color‐changing inks, holograms, watermarks, barcodes, and quick‐response (QR) codes are common examples of anticounterfeiting tags found on various goods. However, since these are produced by deterministic processes, the main barrier to undesired duplication is the lack of access to techniques and materials for the fabrication of the security labels. The widespread access to information in the modern era makes traditional anticounterfeiting tags vulnerable to skilled counterfeiters, with most of the abovementioned deterministic tags being at risk of duplication within 18 months regardless of how sophisticated they are.^[^ ^3^ ^]^ A physical unclonable function (PUF), also called physical one‐way function,^[^ ^4^ ^]^ is considered the most promising solution to the counterfeiting issue. A PUF is a physical object containing fingerprint‐like characteristics with inherent randomness, typically introduced by a stochastic process. Thanks to the use of non‐deterministic preparation methods, PUF devices are typically easy to synthesize and theoretically impossible to copy. PUFs are also an advantageous alternative to software‐based validation systems that can be vulnerable to external intervention such as hacking or electromagnetic interference.^[^ ^5^, ^6^ ^]^ The design of PUF devices involves fabrication, digitization, and authentication. Following structural design and fabrication, the device can be characterized, and the obtained readout is further digitized and stored in the data‐cloud by manufacturers. In this regard, deep‐learning models for feature matching can be introduced to cross‐reference the readout/digitized codes between end‐users and the data‐cloud for authentication.

For each PUF, an input query or “challenge” receives a specific output or “response”, a process known as a challenge–response pair (CRP). The performance of a PUF device in response to a challenge can then be evaluated using a panel of key metrics such as randomness, uniqueness, and repeatability, defined respectively by entropy, inter‐ and intra‐ correlations. For binary codes, bit uniformity measures the probability of observing “1‐bit” or “0‐bit” states in the response bits. An ideal PUF should generate an equal number of “0” and “1” bits (i.e., 50% each) across its responses. Bit uniformity is the primary metric for evaluating randomness in PUFs, ensuring unbiased 0/1 distribution. Entropy complements this by assessing deeper unpredictability. Determining the entropy for all possible challenges provides an estimation of the security level of a PUF, with a high entropy making prediction and forging more difficult for attackers.^[^ ^7^ ^]^ Uniqueness quantifies the ability of a PUF to produce distinct responses across different devices, ensuring that CRPs from separate devices exhibit no statistical correlation. This property guarantees that each PUF generates a unique identifier, enabling reliable differentiation between individual devices. This can be determined using the average inter‐device Hamming Distance (HD) which shows the number of different bits between two individual PUFs. In an ideal scenario, inter‐device HD should equal 0.5, indicating maximum distinctiveness across devices. Repeatability (or reliability) assesses how consistently the same PUF reproduces its response under varying conditions (e.g., temperature, voltage), which ensures stable cryptographic key generation. Determined by measuring the consistency of a single PUF's responses over multiple trials, repeatability is often quantified by the average intra‐device HD (i.e., the bitwise differences between two responses from the same PUF under the same challenge), and should be 0 in an ideal device. Information capacity is another important property as it indicates the robustness of the PUF device. A PUF can be forged when the “fingerprint” is simple enough to be replicated, or when the encryption capacity of the device is not high enough thus making it vulnerable to counterfeiters. The information capacity of a PUF is related to the number of pixels and number of responses of the device, with a suggested minimum value of 10^300^.^[^ ^8^ ^]^ Another important performance indicator is the resistance to machine learning attacks to avoid vulnerability to advanced algorithms modeling and predicting PUF responses. The design of PUF devices must therefore exhibit structural and/or response complexity to retain their “unclonable” nature.

Silicon‐based PUFs, are currently the most well‐developed class of PUFs after having concentrated initial efforts.^[^ ^9^ ^]^ However, the majority of traditional silicon PUFs typically generate only one response per pixel, which limits their capacity and makes them relatively weak. Therefore, efforts have lately shifted toward non‐silicon PUFs, especially those generated by chemical approaches. Chemical PUFs rely on the preparation and encoding of unique nano‐ and micro‐patterns such as random distribution of nanodots or polymer wrinkles.^[^ ^8^ ^]^ The large parameter space in chemical approaches provides high encoding capacities for PUF devices. The key features of general PUF devices (silicon and non‐silicon PUFs) and chemically generated PUFs were discussed more extensively by Abbott^[^ ^9^ ^]^ and Sørensen.^[^ ^8^ ^]^

**Figure 1:**
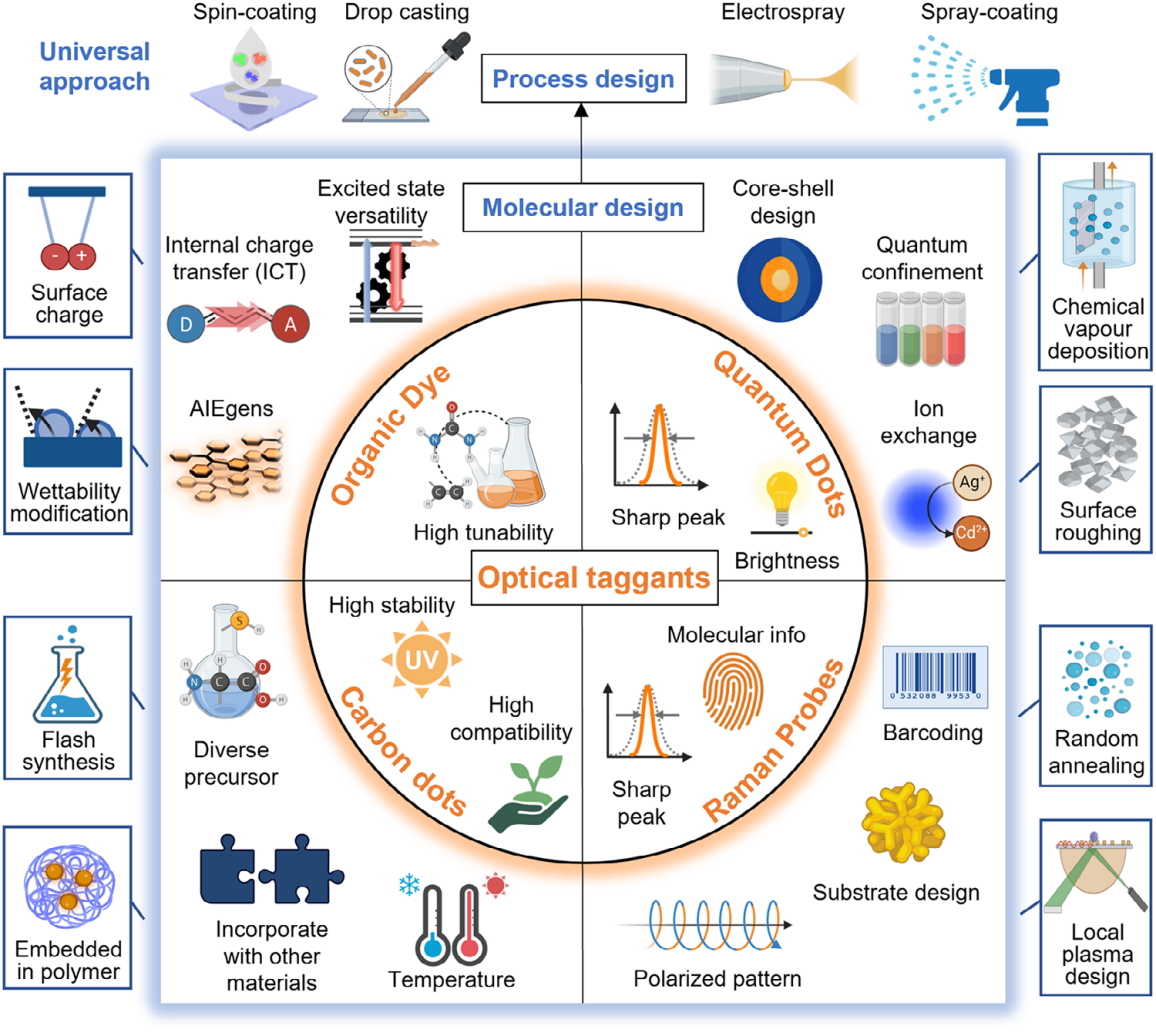
Types of materials and design strategies utilised in the fabrication of PUFs for anticounterfeiting applications.

Optical systems are popular in multiple fields since they can be identified in a non‐invasive and non‐destructive manner. These properties give optical PUF devices particular advantages for readability, stability, and performance in comparison to other chemically generated devices. Optical PUFs are therefore prepared by generating patterns of optically active compounds using stochastic processes. In this review, we aim to provide a guide to the fabrication of PUF devices based on a selection of optical inks, matrices, and manufacturing techniques (**Figure** **1**). To this end, we first present an overview of resonant and non‐resonant optical materials used as security inks in anticounterfeiting applications, then contextualize their use specifically in state‐of‐the‐art PUF devices, with a particular focus on the past 5 years. Within this framework, we aim to provide forward‐looking perspectives, opportunities, and challenges in the young and fast‐paced field of next‐generation optical PUF devices.

## 2 Design of Optical Taggants for Security Inks

### 2.1 Types of Optical Readouts and Key Features of Security Inks

**Figure 2:**
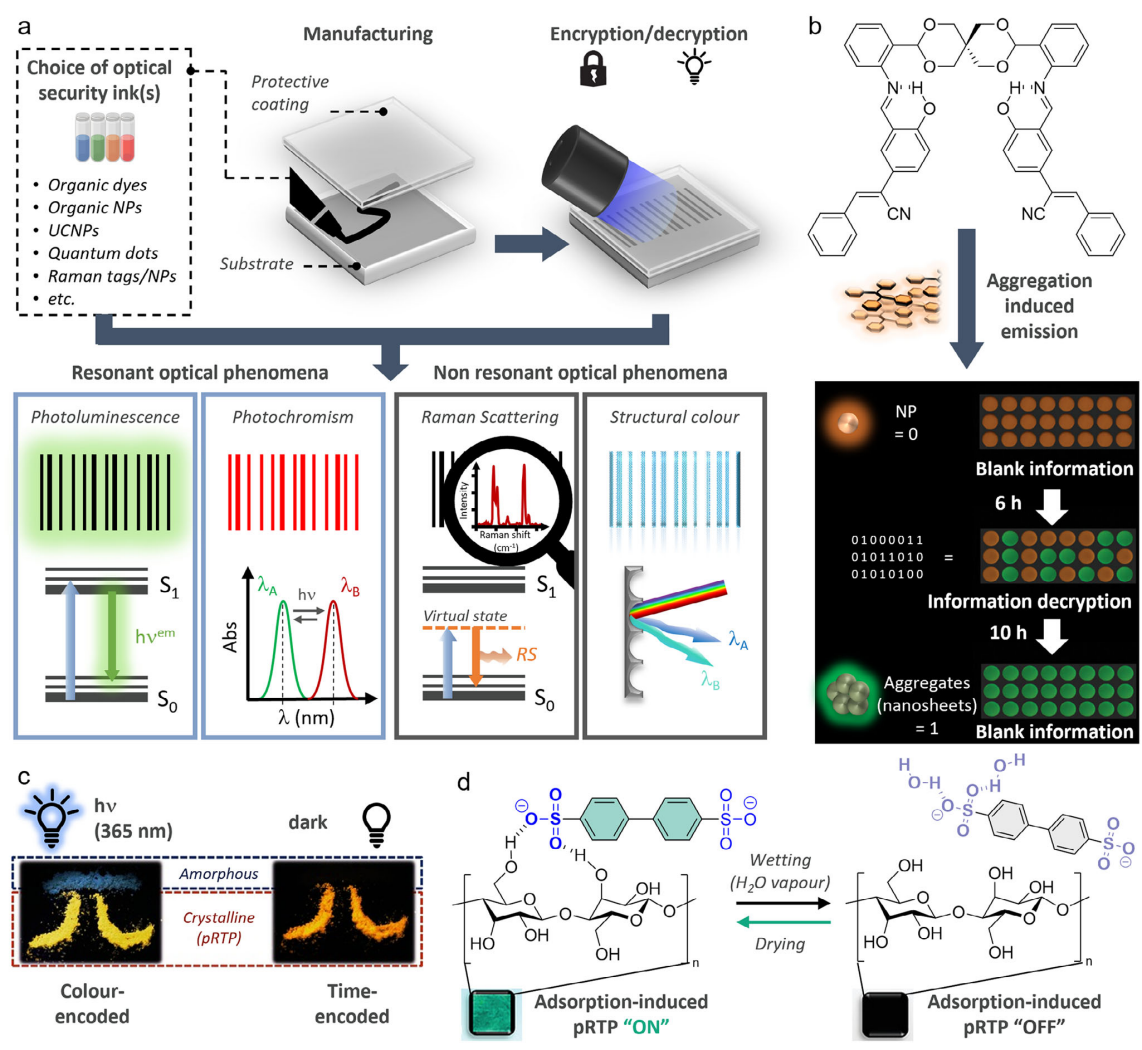
General considerations on the design of anticounterfeiting devices with optical inks, and examples of devices prepared with organic luminophores. a) Optical security devices are prepared by processing optically active inks, that can rely on resonant or non‐resonant phenomena, onto a substrate protected by a matrix or coating. The readout is performed by exposure to light. The type of security is represented here by a barcode for simplicity. b) A cyanostilbene dimer AIEgen can be processed into orange‐emitting NPs that turn into green‐emitting aggregates over time, thereby allowing time‐dependant encryption. Reproduced with permission^[^ ^32^ ^]^ Copyright 2024, John Wiley and Sons. c) Amorphous and crystalline brominated 4‐(9*H*‐carbazol‐9‐yl)benzophenone allow for color‐coded and time‐resolved encryption based on the persistence of their PL after irradiation. Reproduced with permission^[^ ^41^ ^]^ Copyright 2016, John Wiley and Sons. d) Sulphonated bis‐phenyl compound is able to switch‐on pRTP when adsorbed onto a solid surface like weighing paper. The pRTP is switched off when the compound is desorbed from the surface because of hydrogen bonding with water. Reproduced with permission^[^ ^49^ ^]^ Copyright 2023, John Wiley and Sons.

Optical security inks are pigment formulations designed to be visibly read or revealed upon illumination. Their response to light exposure can be divided between resonant and non‐resonant optical phenomena (**Figure** **2**a). In the first category, the readout will be based on i) luminescence (e.g., fluorescence or phosphorescence) or ii) a change in optical properties (e.g., photochromism), whilst, in the second category, the readout will leverage iii) scattering (e.g., Raman spectroscopy) or iv) physical optics (e.g., structural color, nanoscale reflection or diffraction of light, etc.). More sophisticated security devices will combine these phenomena together and generate condition‐dependant readouts to increase the encoding capacity. Importantly, these optical phenomena must be efficient in the solid state or as part of a solid matrix. Application‐based features must also be further considered. The functional pigments may for example need to be resistant to environmental stress such as prolonged light exposure, heat, and mechanical abrasion to preserve security over time. Depending on the final merchandise to be tagged (i.e., packaging, documents, medicine, etc.), the material bio‐ and environmental compatibility or recyclability may need to be considered. Further incorporation into a matrix (nanostructures, polymers, phase‐changing materials, etc.) and use of a protective coating can synergistically improve the abovementioned features (Figure 2a). The processability of the ink is also a key part of the manufacturing of the anticounterfeiting device.

With most optical PUF devices still in the development stage, the perspective of adopting advanced, established, and/or well‐designed security inks has the potential to push the field of unclonable security to the next level. In the next section, we summarize the types of materials commonly reported as security inks and the optical phenomena associated with them, including fluorescence extinction and/or switch on, color changes or visible disappearance upon contact with additional chemicals. Each type of optical material has its own specific features which could influence the performance and practical application of an anticounterfeiting device to a large degree. This section does not aim to present an extensive list of anticounterfeiting inks, but rather a selection of concepts and materials usable for security applications based on recent reports. We refer the reader to other excellent reviews dedicated to security inks for more comprehensive detail.^[^ ^10^, ^11^, ^12^, ^13^, ^14^ ^]^

### 2.2 Photo‐Absorbing Materials Used as Security Inks

#### 2.2.1 Organic Luminophores

Resonant light can trigger a variety of radiative or non‐radiative phenomena in organic dyes. Molecular luminophores undergo radiative relaxation leading to the emission of light from a singlet (fluorescence) or triplet excited state (phosphorescence).^[^ ^15^ ^]^ They have become essential building blocks of light‐responsive inks, whether used alone or in combination with other materials. Organic luminophores are chosen for their well‐defined structures, as well as their tuneable and versatile properties.^[^ ^16^ ^]^ In spite of their formidable mechanistic versatility, some limitations of organic dyes include tedious multi‐step synthesis, necessary when designing custom‐made structures, as well as poor photo‐stability. A wide variety of organic luminophores,^[^ ^10^ ^]^ such as widely available fluorescein and rhodamines^[^ ^17^ ^]^ or custom‐made dyes,^[^ ^18^ ^]^ can be formulated as printing inks or adsorbed onto solid matrices to develop straightforward anticounterfeiting technologies. Red‐green‐blue (RGB) encryption can be easily achieved thanks to this large choice, and basic (e.g., inkjet) or advanced (e.g., laser‐induced forward transfer, LIFT) printing technologies can be leveraged to achieve higher security levels.^[^ ^17^, ^19^ ^]^ Organic luminophores can also be combined with nanoparticles (NPs) to modulate the ink's optical properties.^[^ ^20^ ^]^

One of the key challenges in the use of organic luminophores as security materials is to preserve (or generate) photoluminescence (PL) in solid state and matrices.^[^ ^21^ ^]^ Traditional organic fluorophores, with their planar conformation, are prone to forming stacked aromatic structures leading to aggregation‐caused quenching (ACQ). This limits their PL performance in aggregated or solid state, which is a key issue for luminescence‐based anticounterfeiting devices.^[^ ^22^ ^]^ Aggregation‐induced emission (AIE) and luminescent organic crystals have counteracted this issue by controlling the amount of non‐radiative decay in the solid state.^[^ ^23^ ^]^

AIE luminogens (AIEgens) are dyes that exhibit non‐emissive behavior in solution because of significant molecular motion but become strongly fluorescent upon aggregation.^[^ ^24^ ^]^ Tetraphenylethylene (TPE),^[^ ^25^ ^]^ barbituric,^[^ ^26^ ^]^ pyrene,^[^ ^27^ ^]^ cyanostilbene,^[^ ^28^ ^]^ and carbazole^[^ ^29^ ^]^ derivatives containing known motion‐restricting moieties have been reported, among others, as valuable components of security inks and coatings.^[^ ^21^, ^30^ ^]^ Notably, Zhang et al. recently reported pyrene‐based AIEgens with red emission wavelengths (up to 686 nm) with potential for use in anti‐counterfeiting stamps.^[^ ^31^ ^]^ AIEgens gain even more potential when made time‐ or stimulus‐dependant, thus enabling pluri‐photoresponsive and logic‐gating materials. Time‐dependant encryption was, for example, achieved using a spirocyclic scaffold‐bridged cyanostilbene dimer (Figure 2b).^[^ ^32^ ^]^ Orange‐emitting NPs prepared from these AIEgens turned into green‐emitting nanosheets over time due to supramolecular self‐assembly, with the transformation rate controlled by the water fraction. AIEgens can be covalently or non‐covalently incorporated into polymer matrices to design security devices.^[^ ^11^ ^]^ TPE‐functionalized squaraine AIEgens reported by Yao et al.^[^ ^33^ ^]^ were integrated into an elastomer to create high‐performance thermoresponsive materials, thereby enhancing contrast and encryption. A series of blue and green‐emissive imidazole‐stilbene AIEgens developed by Chen et al. exhibited positive solvatochromism, which led to polarity‐dependant fluorescent security inks.^[^ ^34^ ^]^ A TPE‐based cross‐linker was also used to prepare multi‐stimuli‐responsive polymers for encryption.^[^ ^35^ ^]^ The fluorescence of the materials can be tuned by adjusting the polymer blend composition, thus enhancing the potential for security applications. Finally, AIEgens can exhibit intrinsic or added photochemical behavior (see also next sections).^[^ ^36^ ^]^ The photocyclization and photooxidation properties of TPE were exploited to trigger emission changes in TPE‐pluronic and TPE‐saccharide organic NPs.^[^ ^37^ ^]^

Although challenging, the design of all‐organic materials with persistent phosphorescence in air and at room temperature has been increasingly proposed for security devices.^[^ ^38^ ^]^ Briefly, persistent room‐temperature phosphorescence (pRTP)^[^ ^39^ ^]^ can be achieved in aggregated,^[^ ^40^ ^]^ rigid crystalline^[^ ^41^, ^42^, ^43^ ^]^ or amorphous state,^[^ ^44^ ^]^ and in polymer matrices.^[^ ^45^, ^46^ ^]^ Molecular design strategies involve tuning the intersystem crossing (ISC) in solid state, for instance by modulating intermolecular electronic coupling of *n*π* and ππ* units (e.g., carbonyle and heavy atom halogenated molecules) (Figure 2c),^[^ ^41^, ^42^ ^]^ and by controlling packing density^[^ ^44^ ^]^ and intermolecular interactions.^[^ ^40^ ^]^ Other strategies involve doping rigid organic crystals with iodinated derivatives with halogen bonding capacity to promote spin‐orbit coupling and yield intense pRTP.^[^ ^47^ ^]^ Co‐crystallization of an electron‐donating and ‐accepting molecule can also generate long‐lived charge separation leading to pRTP for controllable pattern anticounterfeiting.^[^ ^48^ ^]^ An elegant example of pRTP relying on restricted thermal motion of sulphonic acids adsorbed onto various matrices rich in hydroxyl groups (paper, cloth, paper money, etc.) was also reported (Figure 2d).^[^ ^49^ ^]^ Melanin was also used as a cheap, widely available organic material for pRTP anticounterfeiting.^[^ ^50^ ^]^ Ultralong lifetimes, mechano‐ and thermally triggered “afterglow” PL have also been achieved for time‐resolved encryption.^[^ ^40^, ^51^ ^]^

A key strategy to increase the potential security level of the ink formulation is to introduce a condition to the organic material's PL. Many intrinsic or synergistic photophysical features can be exploited to achieve this. Solvatochromism and fluorogenicity based on a change in micro‐environment are examples of intrinsic properties that can be leveraged. Fluorophores designed by incorporating boronated dyes as chain extenders into hydrogen bond‐abundant matrices,^[^ ^52^ ^]^ flexible boron‐phosphine oxide Lewis pairs,^[^ ^53^ ^]^ and cyanostilbene‐containing donor–acceptor structures^[^ ^54^ ^]^ show reversible and high‐contrast solvatochromic and fluorogenic properties. Fluorescence switch‐on/off can also be achieved by changes in pH,^[^ ^36^, ^55^ ^]^ heat,^[^ ^56^, ^57^, ^58^ ^]^ or chemical environment (e.g., oxygen,^[^ ^59^ ^]^ ammonia,^[^ ^60^ ^]^ solvent vapors,^[^ ^58^, ^61^, ^62^ ^]^ ions,^[^ ^56^, ^63^ ^]^ etc.), among other conditions.^[^ ^10^, ^64^ ^]^ QR codes were for example printed with a TPE‐diaminotriazine dye combining AIE and pH‐response.^[^ ^36^ ^]^ Temperature‐responsive fluorescent pixel arrays were prepared by incorporating anthracene and perylene derivatives in paraffin phase‐change materials, thereby leveraging ACQ to switch the PL “on” or “off”.^[^ ^57^ ^]^ In an elegant strategy, reactive inkjet printing was used to synthesize a library of luminescent cyanostilbene derivatives from their reactive ink precursors.^[^ ^58^ ^]^ The in situ chemical reactions produced arrays of materials with phase‐, solvent vapor‐ and heat‐dependant PL. Oxygen‐sensitive cyan‐magenta‐yellow (CMY) inks were prepared from porphyrin and naphthalimide fluorescent sensors.^[^ ^59^ ^]^ Amine‐responsive sensors were also developed, for instance by leveraging excited‐state proton transfer in triazole derivatives.^[^ ^60^ ^]^ Combinations of external factors can lead to elegant systems with tuneable emissions.^[^ ^56^ ^]^

Finally, conditional fluorescence can be achieved by introducing energy transfer phenomena, whether with pairs of luminophores^[^ ^55^ ^]^ or photoswitches (see next section).^[^ ^65^ ^]^ Anti‐counterfeiting inks have been developed leveraging Förster Resonance Energy Transfer (FRET) between coumarin‐modified microcrystalline cellulose and fluorescein‐derived poly(amidoamine) (PAMAM) dendrimers for high‐security applications in documents and artworks. The amino‐functionalized dendrimer and pH‐responsivity of fluorescein also added iono‐ and acido‐chromism properties to the system. The variety of photophysical mechanisms involved in organic dyes thus make them key building blocks in the design of security devices.

#### 2.2.2 Organic Photochromic Materials

Photochromic compounds undergo reversible modifications of their π‐conjugated structure upon light stimulation, which causes changes in the optical properties (absorption, color, etc.) of the material. Absorption of resonant light by the initial, thermodynamically stable form of the dye triggers photochemically‐ or thermally‐reversible modifications such as ring‐opening/closing, *cis*/*trans* isomerization, proton transfer, or dimerization reaction, leading to a higher‐energy form. From this, the return to the initial form occurs through thermal processes (T‐type photochromism) or further photoexcitation (P‐type photochromism). Photochromism is typically a unimolecular phenomenon, with positive photochromism occurring when the transformation causes a bathochromic shift in absorption, and negative photochromism representing the opposite case.

**Figure 3:**
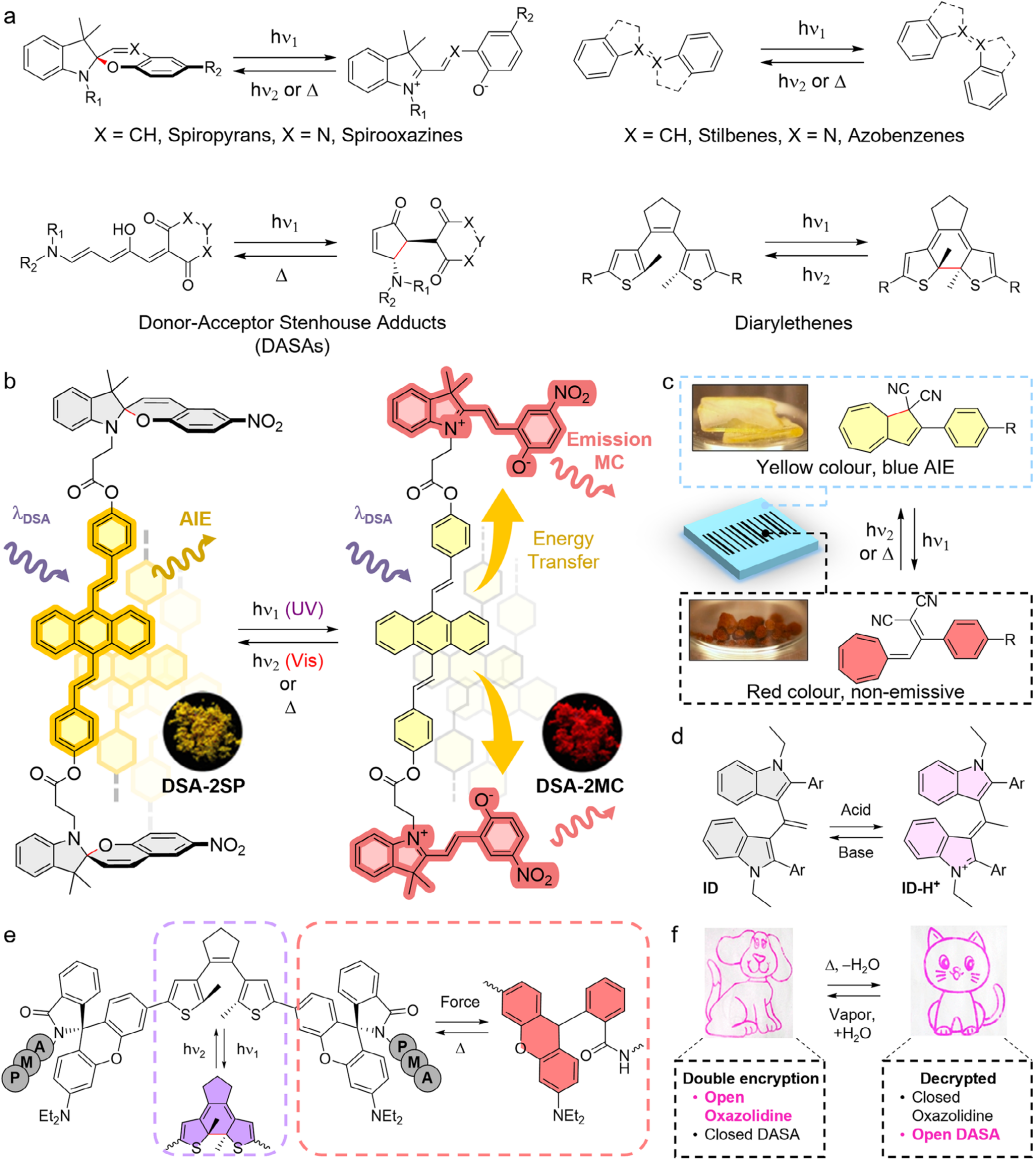
Organic stimuli‐chromic materials commonly used as anticounterfeiting inks. a) Common organic photochromic dyes and their response to the appropriate stimuli. b) Open and closed states of the AIE‐based DSA‐2SP anticounterfeiting ink enabling conditional yellow or red emissions. Pictures of the fluorescent powders were reproduced with permission^[^ ^74^ ^]^ Copyright 2017, American Chemical Society. c) Dihydroazulene‐based security ink engineered for blue AIE and its non‐emissive vinylheptafulvene open state obtained by photochromism. Pictures of the colored powders were reproduced with permission^[^ ^76^ ^]^ Copyright 2022, John Wiley and Sons. d) Indole dimer (ID) displays reversible fluorescence changes when tautomery is promoted by acid/base exposure.^[^ ^90^ ^]^ e) A poly(methylacrylate) (PMA) polymer coupled with a diarylethene/rhodamine dyad as a self‐reporting photochrome mechanophore enabling color switches under force/heat.^[^ ^81^ ^]^ f) An inkjet printed encrypted pattern switching to its “truth” form by hydrochromicity of DASA and oxazolidine inks. Pictures reproduced with permission^[^ ^93^ ^]^ Copyright 2020, American Chemical Society.

These properties enable dual‐security features in anticounterfeiting inks, making photochromic materials valuable for sophisticated security devices that can be dynamically switched between reading states.^[^ ^10^, ^66^ ^]^ Multi‐state devices lead to high encryption capacity, with both overt and covert security elements. Anticounterfeiting inks are typically designed using stimulo‐chromic compounds such as azobenzenes,^[^ ^67^ ^]^ spiropyrans,^[^ ^68^ ^]^ diarylethenes,^[^ ^69^, ^70^ ^]^ and Donor‐Acceptor Stenhouse Adducts (DASAs)^[^ ^71^, ^72^ ^]^ (**Figure** **3**a). Recent highlights of photochromic ink designs include the work by Shi et al. who developed anti‐counterfeiting materials by incorporating spiropyran and Zn^2^⁺ ions into a chiral liquid crystal (LC).^[^ ^73^ ^]^ Multi‐state encryption was achieved via reflective color, fluorescence, and circularly polarized luminescence. Photochromic compounds are often introduced to add a logic‐gate feature to multi‐component systems, particularly via energy transfer mechanisms. A representative example of ink design was reported by Qi et al.^[^ ^74^ ^]^ The authors prepared a distyrylanthracene AIE moiety functionalized with two spiropyrans (DSA‐2SP), therefore giving an ink with yellow AIE in its closed form and FRET‐promoted red emission in its open merocyanine form (Figure 3b). Interesting properties were reported in thin films and poly(methyl methacrylate)‐doped films, such as efficient switchable luminescence with persistence of the merocyanine form for >24 h, and resistance to fatigue, thus showing the potential of photochromes for anticounterfeiting devices. A sensitizing strategy was also reported recently by Lei et al. with a phosphorescent Pt(II)‐spiropyran complex.^[^ ^75^ ^]^ Conditionally fluorescent organogels were also designed for dynamic anticounterfeiting by Le et al. by coupling spyropyrans and naphthalimides via energy transfer in polymer networks.^[^ ^65^ ^]^ The morphology changes of the switch in the solid state can also be directly leveraged in the ink design. The Tang group developed an ink based on the particular properties of dihydroazulene, which can be converted into vinylheptafulvene by light, heat, and Lewis acids, and was further engineered for blue AIE in the closed state. This could be used to fabricate anticounterfeiting patterns with conditional fluorescence (Figure 3c).^[^ ^76^ ^]^ Azo derivatives were also used to generate polarization‐dependent upconversion luminescence thanks to photo‐switchable orientation.^[^ ^77^ ^]^

Importantly, many other stimuli can be used to generate an optical readout from organic stimulo‐chromic materials, leading either to color or PL switches (Figure 3d–f). Mechanochromic materials can be stimulated by drawing or grinding,^[^ ^78^, ^79^, ^80^, ^81^ ^]^ stretching,^[^ ^82^ ^]^ shearing,^[^ ^83^ ^]^ or pressing,^[^ ^84^, ^85^, ^86^ ^]^ which causes transformations within crystalline,^[^ ^85^, ^87^ ^]^ liquid‐crystalline solid‐state materials,^[^ ^83^, ^88^ ^]^ or chromophore‐doped polymers.^[^ ^82^, ^89^ ^]^ Many smart pH‐responsive stimulo‐chromic materials have also been obtained by introducing acid/alkali‐sensitive functional groups (e.g., tautomerisable indole dimers (ID),^[^ ^90^ ^]^ Figure 3d) and chromophores (e.g., pH‐responsive xanthenes,^[^ ^81^ ^]^ Figure 3e). Moreover, hydrochromic,^[^ ^91^, ^92^, ^93^, ^94^ ^]^ electrochromic,^[^ ^95^ ^]^ ionochromic^[^ ^95^ ^]^ and multi‐stimulochromic^[^ ^96^ ^]^ organic dyes have been used as security inks. DASA and oxazolidines have for instance been used for dual hydrochromic encryption (Figure 3f).^[^ ^93^ ^]^ Photocatalytic systems promoting discoloration of redox‐sensitive dyes were also reported.^[^ ^97^ ^]^ This unmatched versatility therefore provides exciting multiplexing combinations, whereby adding several chromophores per material would increase the device security. Photoswitchable devices may however be susceptible to fatigue and degradation after repeated stimulation, which may be a limitation when harsh conditions are involved.^[^ ^98^ ^]^

#### 2.2.3 Conjugated Organic Nanomaterials

##### Conjugated Polymers (CPs)

**Figure 4:**
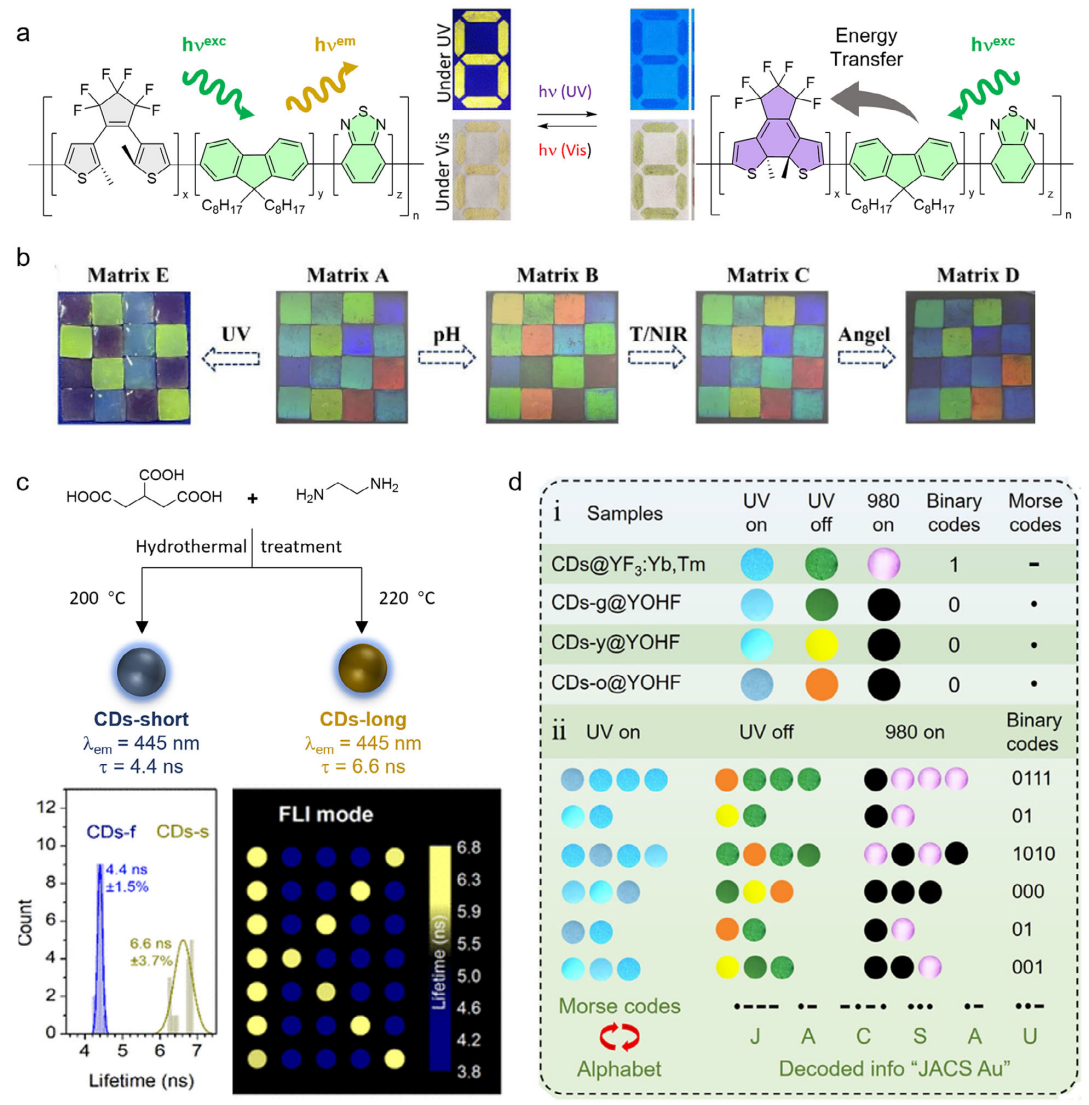
Security inks based on conjugated organic materials. a) Semiconducting polymer chains modified with a diarylethene photochromic unit enable conditional fluorescence from the polymer dot leading to dual‐mode encryption. Photographs reproduced with permission^[^ ^104^ ^]^ with permission. Copyright 2023, Royal Society of Chemistry. b) A multi‐responsive CD/hydrogel matrix, generated by the self‐healing of individual squares. Reproduced with permission^[^ ^113^ ^]^ Copyright 2022, Elsevier B.V. c) CDs synthesized by hydrothermal treatment of citric acid and ethylene diamine at different temperatures exhibit distinctive and well‐separated fluorescence lifetimes enabling lifetime‐encoded security patterns. Reproduced under the terms of the ACS author choice Open Access license ^[^ ^114^ ^]^ Copyright 2018 the Authors. Published by the American Chemical Society. d) Information encoding/decoding process enabled by triple‐mode luminescent lanthanide‐doped CD composite materials. Reproduced under the terms of the CC‐BY‐NC‐ND 4.0 license ^[^ ^116^ ^]^ Copyright 2023 The Authors. Published by American Chemical Society.

Besides small molecules, organic semiconductors are also highly attractive materials due to their synthetic accessibility, high photostability, and flexibility for structural design.^[^ ^99^ ^]^ They consist of large π‐conjugated domains generating continuous electronic band structures in their energy levels. Among them, conjugated polymers (CPs) are important constituents of processable and stimulus‐responsive security inks. Polydiacetylenes (PDAs) are an important type of CP that generates fluorescence in response to environmental stimuli. They can be synthesized by self‐assembly of monomers without the need for initiators or catalysts. Lee et al. patterned PDA by dithering mask lithography, achieving three modes of holograms in a single architecture through solvent exchange.^[^ ^100^ ^]^ CPs can also be formulated by straightforward mini‐emulsion or reprecipitation methods, generating NPs with single particle brightness higher than quantum dots (QDs).^[^ ^101^, ^102^ ^]^ Decorating the backbone of CPs with varied pendant side chains is a convenient way to functionalize these NPs.^[^ ^103^ ^]^ In another example of CP ink, Fang et al. synthesized two photoswitchable semiconducting polymers by incorporating photochromic dithienylethene (DTE) into the main chains (**Figure** **4**a).^[^ ^104^ ^]^ Dynamic pattern encoding was achieved by reversible fluorescence and chrominance switch. In comparison to covalent modification, supramolecular strategies typically lead to less complex preparation and allow the generation of larger libraries of photoelectric materials, which constitutes another appealing approach. Wang and his team developed multi‐responsive CP NPs through supramolecular host–guest assembly.^[^ ^105^ ^]^ The photoresponsive energy acceptor DTE derivative was spatially positioned in the vesicles by self‐sorting encapsulation. The obtained NPs show long‐lasting chemiluminescence and reversible photoswitching between emission and quenching. Chan et al. synthesized two types of photoswitchable polymer dots by incorporating photochromic spiropyran into CPs with covalent and non‐covalent modifications.^[^ ^106^ ^]^ They observed higher photoswitching efficiency of covalently modified NPs as compared to the physically blended ones.

##### Carbon Dots (CDs)

CDs are another type of nanomaterial containing large π‐conjugated domains. They have been applied in multiple fields including biomedicine, energy, and photocatalysis. Although less bright, defined and narrow‐banded than inorganic QDs,^[^ ^107^ ^]^ CDs possess the advantage of being highly accessible by different synthesis approaches using abundant, low‐cost, and even biomass precursors. Their PL is tuneable by doping their π‐conjugated systems with heteroatoms, and they are reportedly biocompatible and photostable. Because of their soft amorphous character, CDs may suffer from *π*–*π* stacking and ACQ in the solid state,^[^ ^108^ ^]^ which hinders direct use as security devices. Doping CDs into matrices (for example, polymers, salts, or starch)^[^ ^109^, ^110^ ^]^ or synthesizing self‐dispersive CDs (e.g., by steric hindrance, electrostatic repulsion, or hydrogen bonding)^[^ ^111^, ^112^ ^]^ are state‐of‐the‐art strategies to achieve solid‐state fluorescence. For example, Yi et al. reported multiple stimuli‐responsive graphene QDs doped in self‐healing hydrogels for information encoding and encryption.^[^ ^113^ ^]^ Dual‐responsive precursors, self‐healing precursors, and CDs were introduced into a hydrogel framework through a two‐step filling method, producing a self‐healing hydrogel with fluorescent properties and multi‐responsive structural color. By changing the size of the NPs in the hydrogels, square fragments with different optical performances could be prepared. Benefiting from the self‐healing property of the hydrogels, these squares adhered tightly when put together at room temperature. The obtained matrix proved responsive to UV, pH, temperature, and the angle of incident light (Figure 4b). Lifetime‐encoded inks have also been developed using CDs. Zbořil et al. prepared CD inks with identical steady‐state emission properties, but distinctive fluorescence lifetimes, proving the concept (Figure 4c).^[^ ^114^ ^]^ More recently, metal‐free CDs@SiO_2_ composites with ultralong pRTP lifetime of 5.72 s (more than 40 s to the naked eye) have been developed thanks to multi‐confinement effects, showing greater potential for the design of lifetime‐encoded inks.^[^ ^115^ ^]^ Moreover, the phosphorescence of the CDs can be tuned by integrating other materials such as lanthanide ions. Zhang et al. incorporated CDs into a YF_3_ matrix doped with Yb^3+^ and Tm^3+^ ions for a triple‐mode luminescence system (fluorescence, pRTP, and upconversion PL, Figure 4d).^[^ ^116^ ^]^ Triple mode luminescence was also exploited by Jiang et al. using a CD/poly(vinyl alcohol) (PVA) composite ink exhibiting PL, upconversion PL, and pRTP. This unique multimodal feature helped raising the technical barriers to counterfeiting.^[^ ^117^ ^]^

#### 2.2.4 Inorganic Materials

##### Rare‐Earth Materials

Rare earth elements constitute very versatile materials exhibiting unique PL properties in the form of ions, complexes, crystals, and nanomaterials. They typically feature UV‐centered, narrow absorption bands and emit visible light with large Stokes shifts, allowing the design of invisible security marks with enhanced detection sensitivity. Their long‐lived excited states and phosphorescence properties, enabled by the heavy atom effect, unlock powerful time‐gated detection techniques with minimal background interference and temporal encoding.^[^ ^118^ ^]^ Lanthanide ions were reported to exhibit dynamic PL properties (e.g., time‐dependant emission) when incorporated in materials containing traps and cross‐relaxation.^[^ ^119^ ^]^ Combining lanthanide ions with organic ligands and within other lanthanide‐containing materials allows to tune their optical properties, achieving for instance multi‐colored emissions via intricate energy transfer mechanisms. As such, one of their most notable features is their ability to upconvert low‐energy excitation (e.g., near‐infrared) photons into high‐energy (e.g., visible) PL photons.^[^ ^120^, ^121^ ^]^ Upconversion nanoparticles (UCNPs) typically consist of a pair of Ln^3+^ ions, using for example Yb^3+^ as a sensitizer and a fine‐tuned ratio of Er^3+^, Ho^3+^, or Tm^3+^ as emitters. Fluoride‐based matrices such as AREF_4_ materials (where A is an alkali metal such as Li, Na, or K, and RE is a rare earth element such as Y, Lu, or Gd) are considered suitable hosts thanks to low non‐radiative relaxation leading to efficient PL.^[^ ^118^, ^122^ ^]^ Lanthanide UCNPs provide exceptional structural and mechanistic versatility, in addition to being compatible with colloidal ink formulations, which represents an attractive option for optical security inks.

**Figure 5:**
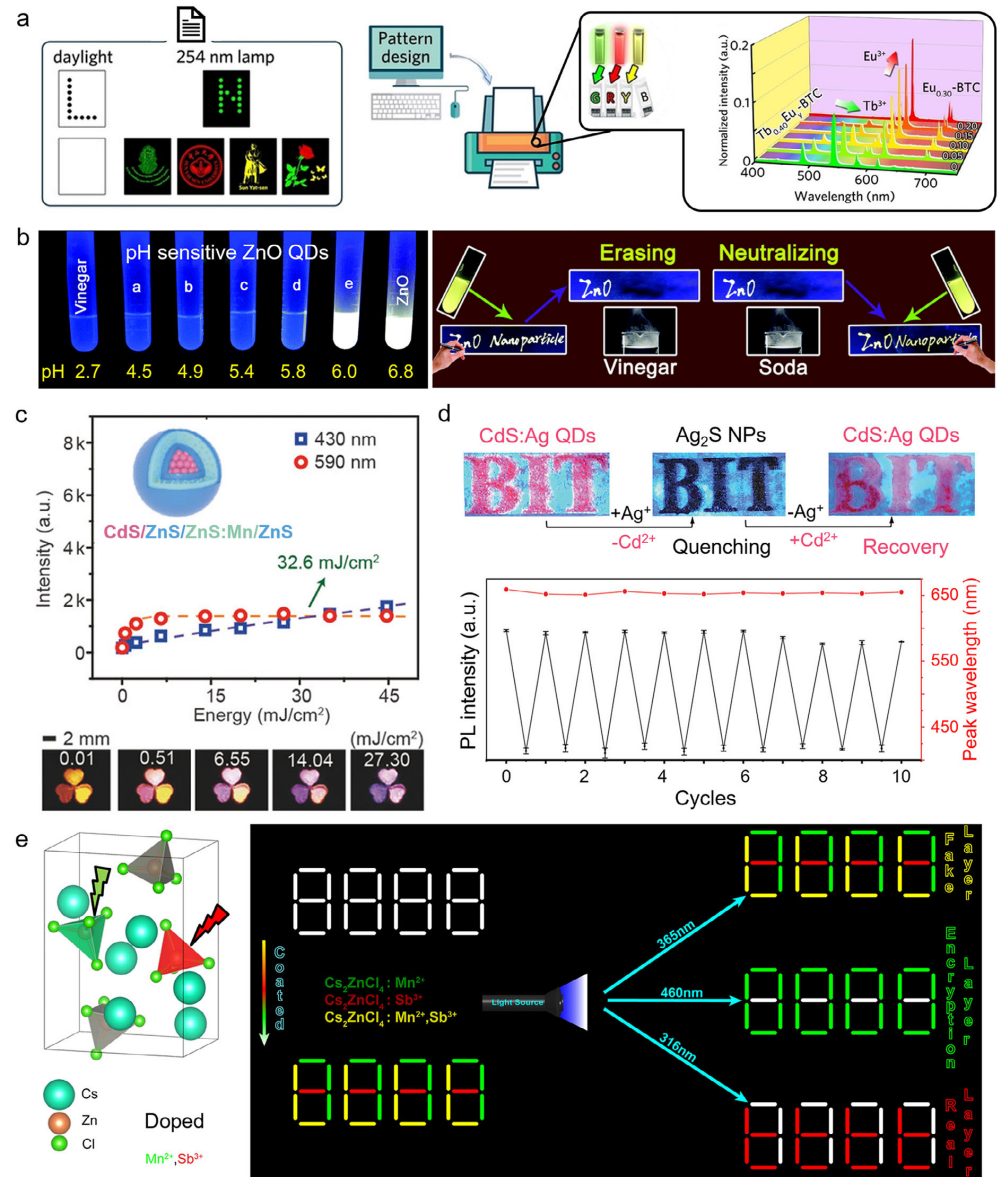
Rare‐earth and QDs designed as security inks. a) Green, red, and yellow photoluminescent Tb_x_Eu_y_‐benzene tricarboxylic acid polymer security inks were used for pattern encryption in the spatial dimension thanks to computer pattern design and inkjet printing. The insert shows the area‐normalized emission spectra (λ_ex_ = 300 nm) of the inks at room temperature. Reproduced with permission^[^ ^124^ ^]^ Copyright 2020, John Wiley and Sons. b) The fluorescence of surface‐modified ZnO QDs can be quenched by acidification, which offers suitable inks for erasable security devices. Reproduced with permission^[^ ^140^ ^]^ Copyright 2017, Royal Society of Chemistry. c) QDs with a core‐multishell structure (CdS/ZnS/ZnS:Mn/ZnS) show both excitation wavelength‐ and power‐dependant PL, which allows the preparation of fluorescent patterns with tuneable colors. Reproduced with permission^[^ ^142^ ^]^ Copyright 2017, John Wiley and Sons. d) Fluorescent patterns of CdS:Ag QDs can be quenched and recovered by in situ ion exchange. Only minor intensity degradation and no significant peak shift were observed after ten quenching and recovery cycles. Reproduced with permission^[^ ^146^ ^]^ Copyright 2019, John Wiley and Sons. e) Perovskite crystals of Cs_2_ZnCl_4_ and Mn^2+^, Sb^3+^ codoped Cs_2_ZnCl_4_ used for wavelength‐dependant information encryption on an “8888” pattern. The real information “tttt” (red PL) is only revealed by exposure to 316 nm light. Reproduced with permission^[^ ^154^ ^]^ Copyright 2023, American Chemical Society.

The versatility of lanthanide materials extends to their compatibility with various substrates, coatings, and processing methods. Inkjet printing of NIR‐responsive UCNP inks was recently exemplified by You et al.^[^ ^123^ ^]^ to print 2D patterns revealed with NIR light, and generate multi‐layered encryption in combination with downconversion dyes. Printable lanthanide‐coordinating benzene‐tricarboxylic acid polymers with high quantum efficiencies tuneable colors and submillisecond lifetimes were also reported by Ou et al. (**Figure** **5**a).^[^ ^124^ ^]^ UCNPs were also dispersed in hydrogels^[^ ^125^ ^]^ and varnishes^[^ ^118^ ^]^ to generate RGB‐ or NIR‐encoded devices. Luminescent hydrogels and polymer films were prepared by complexation of a tetracarboxylic acid ligand with various lanthanide ions, leading to multi‐colored inks with PL in the RGB range.^[^ ^126^ ^]^ Moreover, carboxylic acid ligands can produce pH‐responsive lanthanide complexes, thus adding another layer of security.^[^ ^127^ ^]^ Interestingly, core‐shell NaYF4:Yb,Er NPs with tuneable PL lifetimes (3.2‐6 ms) and stable PL in the short‐wave infrared (≈1530 nm) were reported to enable high‐speed temporal decoding by rapid lifetime determination.^[^ ^128^ ^]^ Thus, the ability to fine‐tune optical properties through dopant selection, concentration, and NP design enables the creation of unique optical “fingerprints”.^[^ ^129^ ^]^ Thanks to these unique and versatile properties, rare earth materials can prove even more valuable as switchable and/or multi‐component systems.^[^ ^130^ ^]^ Yang et al. reported dynamic anticounterfeiting inks embedding spiropyrans into lanthanide metal‐organic frameworks (MOFs), which led to reversible PL modulation through an energy transfer mechanism. Their SP@Ln‐MOF/polydimethylsiloxane (PDMS) films offered high‐contrast, rapid, and reversible color changes, while maintaining flexibility and mechanical strength.^[^ ^131^ ^]^ Li et al. used a combination of photochromic diarylethenes and Eu^3+^‐coordinating polyelectrolytes to generate smart security inks.^[^ ^132^ ^]^ The emission of Eu^3+^ overlaps perfectly with the closed form of the diarylethene, which allows FRET‐based conditional luminescence. These features highlight the fascinating potential and versatility of rare earth materials for anticounterfeiting devices.

##### Semiconductor QDs

QDs are crystalline semiconducting materials of nanometer size (2–20 nm) typically composed of II–VI, III–V, and IV–VI elements.^[^ ^133^ ^]^ They exhibit size‐dependent absorption and emission thanks to the quantum confinement effect, achieving optical properties from the ultraviolet to the near‐infrared (NIR) spectrum,^[^ ^134^, ^135^ ^]^ One key feature is their narrow emission bandwidths, with monodisperse colloidal QDs approaching single‐dot linewidth (20 to 80 meV)^[^ ^136^, ^137^ ^]^ and enabling high color purity. Chromatically pure and orthogonal QDs have been used in multiple commercial applications in lasers, displays, biotags, and solar harvesting devices.^[^ ^12^ ^]^ This chromatic purity also makes QDs ideal optical tags for security inks by allowing wavelength multiplexing and higher encoding capacity. Wet‐chemical approaches and physical‐based epitaxy methods are the main strategies to synthesize QDs. Generally, chemical approaches involve nuclear generation, growth of NPs, and stabilization with surfactants or ligands.^[^ ^138^ ^]^ Chemical approaches are cost efficient and commonly used, while physical methods allow direct integration in a crystallized surface, providing high optical quality and epitaxial heterostructure. Both commercially available and tailored functionalized QDs can be used to prepare inks for security devices. The main strategy applied to develop QD‐based security inks is to make their luminescence condition‐dependant. ZnO QDs are typical probes for erasable devices as their emission is quenched by acid (Figure 5b).^[^ ^139^, ^140^ ^]^ After neutralizing the acid, new information can be written in the same device. Growing epitaxial layers of inorganic material over the QD core was reported to yield core‐shell QDs with improved PL.^[^ ^141^ ^]^ As such, Li et al. designed color‐tuneable CdS/ZnS/ZnS:Mn^2+^/ZnS QDs with a core/multishell structure (Figure 5c).^[^ ^142^ ^]^ The band‐edge emission of the core shows blue PL,^[^ ^143^, ^144^ ^]^ while the ^4^T_1_ → ^6^A_1_ emission of the Mn^2+^ ion in the shell shows orange PL.^[^ ^145^ ^]^ The emission ratio was dependent on the power density of the excitation laser and the concentration of QD. By changing these two parameters, patterns with different fluorescent colors can be prepared. In addition, Zhang et al. reported Ag‐doped CdS QDs enabling dual mode anticounterfeiting by reversible cation exchange (Figure 5d).^[^ ^146^ ^]^ After the addition of methanolic Ag^+^ solution to the CdS:Ag QDs, non‐luminescent Ag_2_S NPs were formed. The fluorescence could be restored by the addition of Cd^2+^ and tributyl phosphine, thereby converting the Ag_2_S NPs into the initial CdS:Ag QDs.

##### Perovskites Quantum Dots (PQDs)

As an alternative to conventional QDs, perovskites have spurred intense research efforts in recent years thanks to their unique properties. The ideal perovskite has a cubic structure with a chemical formula of ABX_3_, where A is an organic or inorganic cation, B is a divalent metal ion, and X is a halogen element.^[^ ^147^ ^]^ Nanosized perovskite materials also exhibit key quantum confinement properties^[^ ^148^ ^]^ and tuneable PL maxima achieved through halide composition engineering.^[^ ^149^ ^]^ Meanwhile, PQDs have high defect tolerance which achieves efficient PL emission without a sophisticated core–shell structure.^[^ ^150^ ^]^ PQDs can be combined with other functional materials or doped with additional ions for stimulus‐dependant emissions. By loading CsPbBr_3_ (CPB) onto electrophoretic particles, Yang et al. developed an anticounterfeiting device with dynamic modulation.^[^ ^151^ ^]^ In situ crystallization of PQDs in glass by femtosecond laser can generate a linear array structure, which contributes to polarization‐sensitive patterns.^[^ ^152^ ^]^ Different PQDs were also reported to form multilayer composites.^[^ ^153^ ^]^ The encrypted information is patterned in one PQD layer and concealed by another PQD layer with distinct PL peak positions, temperature dependences and PL lifetimes. Low dimensional perovskite single crystals doped with metal cations were also used as multi‐mode anticounterfeiting reporters, revealing information only upon exposure to a specific wavelength of UV light (Figure 5e).^[^ ^154^ ^]^ Importantly, PQDs are highly compatible with lanthanide‐doped UCNPs, thus producing anti‐Stokes upconverted PL under NIR excitation. Composites of UCNPs and PQDs display long‐afterglow luminescence,^[^ ^155^ ^]^ and UV/IR/thermal responsive multicolor fluorescence for multimode anti‐counterfeiting.^[^ ^156^, ^157^, ^158^, ^159^ ^]^  Despite the superior optical properties of PQDs, their inherent structural instability may pose significant barriers to practical application. Strategies such as silica coating,^[^ ^160^ ^]^ encapsulation in polymers,^[^ ^161^ ^]^ or mesoporous matrixes^[^ ^162^ ^]^ were reported to enhance the stability of PQDs.

### 2.3 Non‐Resonant Optical Materials Used as Security Inks

#### 2.3.1 Raman Probes

Though fluorescence encoding is popular due to its high brightness and easy readout, it typically allows limited multiplexing capacity because of broad emission bandwidth. Raman scattering exhibits much narrower peaks (≈10 cm^−1^), which provides intrinsic potential for advanced encryption and higher encoding capacities. Raman scattering is the inelastic scattering of photons by molecules. The energy difference between incident and scattered photons corresponds to specific molecular vibrational states, which generates spectra that display scattering intensities as a function of Raman shifts. Raman signals, especially surface‐enhanced Raman scattering (SERS),^[^ ^13^ ^]^ constitute molecular “fingerprints” that can be used for identification and analysis, warranting extensive use as a characterization technique, but also for bio‐imaging or in biosensors.^[^ ^163^ ^]^ Beyond such uses, SERS is also particularly adapted to security applications that intrinsically rely on surfaces and/or nanopatterns. Gold and silver nanomaterials are the most popular SERS substrates for anticounterfeiting since they give giant physical enhancement (up to 10^14^) when in close proximity with the reporter,^[^ ^164^, ^165^ ^]^ and their cost is relatively trivial compared to the overall expense. Associated to the plasmonic material, molecular reporters should have strong adsorption (physical or chemical) onto the plasmonic material, a low number of vibrational peaks to avoid signal overlapping, and high stability.^[^ ^13^ ^]^ Thiolated reporters are commonly used to allow convenient functionalization of gold plasmonic surfaces. Commercially available organic dyes (e.g., Rhodamine 6G) are also widely used, although PL can be a source of high background noise and poor readout. Layered materials, such as “core‐gap‐shell” NPs have also attracted interest to amplify plasmonic enhancement between layers. Embedding Raman‐active reporters in the gap of such materials has led to the development of a specific type of Gap‐enhanced Raman tags (GERTs).^[^ ^166^ ^]^ Notably, some molecules show high signal‐to‐noise ratio in the so‐called silent region of the Raman spectrum (1800–2600 cm^−1^), where only a handful of functional groups show vibrational signals (e.g., alkynes, nitriles, azides etc.).^[^ ^167^ ^]^ Raman reporters in this region would not interfere with the signals of other matrix or support materials, offering another choice for advanced anticounterfeiting devices.

**Figure 6:**
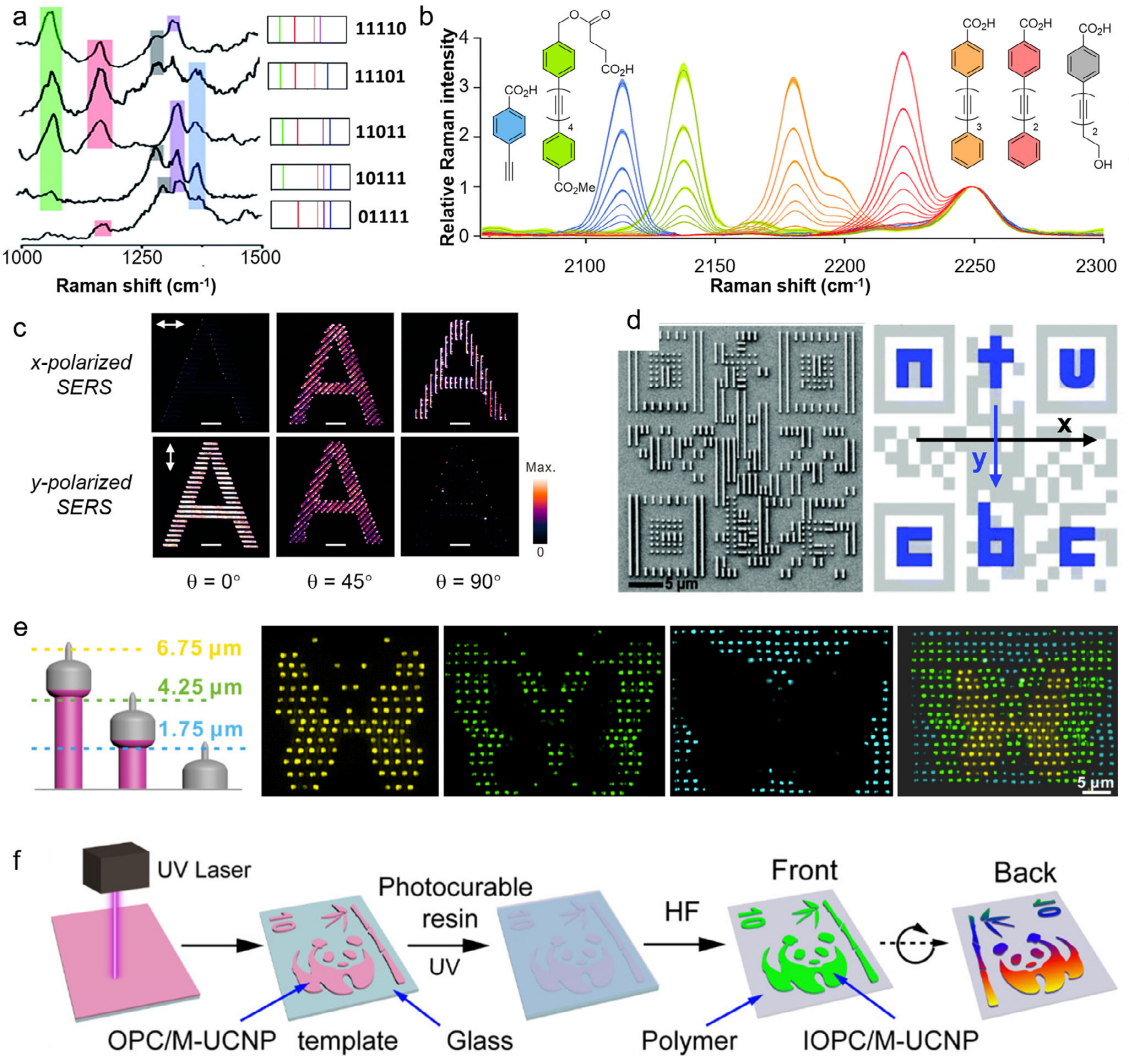
Anticounterfeiting labels developed with Raman reporters and structural color materials. a) Example of barcode encoding generated from the orthogonal Raman signals of multiple reporters. Reproduced under the terms of the CC BY‐NC 3.0 license ref. [^168^ ^]^ Copyright 2015, The Authors. Published by the Royal Society of Chemistry. b) Example of alkyne Raman tags generating distinct spectral bands with nonoverlapping characteristic peaks in the silent region. Encoding was performed by 8 different dosages of each Raman tag. Reproduced under the terms of the CC BY‐NC 3.0 license ref. [^168^ ^]^ Copyright 2020, The Authors. Published by the Royal Society of Chemistry. c) x‐ and y‐polarized 2D SERS images of Ag nanowires generating an “A” pattern at different orientation angles. Reproduced with permission^[^ ^170^ ^]^ Copyright 2014, American Chemical Society. d) Two‐tier security label prepared from anisotropic polarisation‐dependant SERS‐active aluminum nanostructures generating a QR code overlaid with ciphertexts. Left picture shows the scanning electron microscopy (SEM) image of the label, and right image shows the QR code (grey) and ciphertext (blue) embedded in the label. The axes show the polarisation. Reproduced with permission^[^ ^171^ ^]^ Copyright 2018, Royal Society of Chemistry. e) Three layers of patterns are decrypted by extracting individual x–y SERS images at the corresponding z values of 6.75, 4.25, and 1.75 µm. Reproduced with permission^[^ ^172^ ^]^ Copyright 2017, American Chemical Society. f) Preparation of three patterns (“10” “bamboo”, and “panda”) with inverse opal crystal films doped combined with green‐emissive UCNPs allowing triple anticounterfeiting based on reflection, scattering, and PL. Reproduced with permission^[^ ^177^ ^]^ Copyright 2022, American Chemical Society.

The design of SERS inks depends on the type of information used for encoding (i.e., SERS spectra or SERS imaging). Characteristic peaks and peak intensities of the Raman SERS spectra can be digitized as barcodes for anticounterfeiting (**Figure** **6**a).^[^ ^168^ ^]^ Due to the low complexity and low encoding capacity of barcodes, multiple Raman reporters are typically combined to increase the security level. As such, Gao et al. combined distinct spectral bands and intensity levels to realize super‐capacity information‐carrying systems.^[^ ^169^ ^]^ Alkyne and oligo‐yne Raman tags presenting 4 distinct spectral bands were employed in their work (Figure 6b). Silent region reporters were used to avoid peak crowding and interference of signals in the fingerprint region (500–2000 cm^−1^). Every compound maintained a specific Raman shift and allowed for the design of octal code units, thanks to 7 tag dosages, thus offering high information capacity. In Raman imaging, images are reconstituted from both spectral and spatial information by composing each pixel with a complete Raman spectrum. Therefore, anti‐counterfeiting labels based on Raman imaging have higher security potential than those based on Raman spectra. Meanwhile, SERS images can be combined with polarized light, which brings extra encryption. For example, by manipulating the orientation of nanowire nanostructures, Ling's group achieved quantitatively tuneable SERS signals (Figure 6c).^[^ ^170^ ^]^ Lay et al. also fabricated miniature security labels with a polarization‐dependent SERS response.^[^ ^171^ ^]^ A machine‐readable QR code and a set of ciphertexts could be discovered when scanning the label in x‐ and y‐polarized orientations, respectively (Figure 6d). Moreover, z‐axis‐dependent SERS readouts were introduced to extend “layered security” capabilities from two to three dimensions, further increasing the encoded data in the same area (Figure 6e).^[^ ^172^ ^]^

#### 2.3.2 Other Non‐Resonant Phenomena

Structural color has been a common non‐resonant optical phenomenon used in anticounterfeiting tags,^[^ ^14^ ^]^ for example with optically variable inks used on banknotes. It arises from light interference in submicrometer periodic structures, fine enough to reflect specific wavelengths of light. This phenomenon, responsible for numerous colored patterns in nature, does not require pigments. Instead, structural color devices leverage manufacturing techniques (lithography, two‐photon polymerization, 3D printing, etc.) to fabricate patterned polymer and colloidal materials such as photonic crystals, LCs, films, and photonic glass. Thanks to high processability, tunability, and stability, structural color materials have achieved commercial success in hologram tags. A complex array of optical parameters (e.g., angular dependence, polarisation dependence, reflectance maxima, etc.) can be harnessed for high encryption capacity. Structural color materials can be prepared using polymer colloids. Among recent examples, Li et al. reported a rapid and scalable method to produce iridescent films composed of carboxylated polystyrene (PS) colloids ordered in a poly(ethyleneimine) (PEI) polymer matrix.^[^ ^173^ ^]^ The supramolecular and electrostatic interactions between the components generated an ordered colloidal arrangement under shearing responsible for structural coloration. Anticounterfeiting inks generating structural color have also been prepared using layered thin films of Ag and SiO₂ layers leveraging coupled cavity resonance.^[^ ^174^ ^]^ Cholesteric LCs have also been proposed as Bragg reflectors in anticounterfeiting devices.^[^ ^175^, ^176^ ^]^

Reflection of light can be combined with resonant materials for multi‐layered encryption. Meng et al. deposited UCNPs on a polymer bilayer inverse opal photonic crystal film with photonic stop band.^[^ ^177^ ^]^ The structural colors of the photonic crystals were observed in specular and nonspecular angles, and the photonic stop bands, matching the excitation of the UCNPs, enhanced luminescent readout to enable triple anticounterfeiting (Figure 6f). Monodispersed poly(ethyl acrylate–styrene–divinylbenzene) microspheres were also combined with NaYF_4_:Er^3+^,Yb^3+^ UCNPs to produce anticounterfeiting films exhibiting angle‐dependent structural color and PL.^[^ ^178^ ^]^ In another example, Han et al. reported a rewritable PL and structural color display for dual optical encryption using a poly(styrene‐block‐2‐vinylpyridine) (PS‐*b*‐P2VP) block copolymer photonic crystal in which luminescent perovskite nanocrystals were preferentially self‐assembled. Other non‐resonant optical phenomena have been used to produce physical color or alterations in optical properties,^[^ ^179^ ^]^ which includes for example holograms,^[^ ^180^ ^]^ and changes in material transparency.^[^ ^181^ ^]^ Some have been used for the fabrication of PUF devices as detailed next.

## 3 Design and Fabrication Strategies for PUF Devices

To develop a PUF device from an optical probe, it is critical to make the probe compatible with a stochastic process. Specifically, the patterns generated by the stochastic process should be truly random and unique, with the ink remaining stable and showing high performance after patterning. Their randomness can be assimilated to and quantified by the entropy of the system.^[^ ^7^ ^]^ Other elements to consider are the measurement techniques, encoding methods, and authentication approaches. The measurement techniques need to be effective and efficient to capture the optical responses of PUFs. Exclusive techniques and those with long acquisition times should be avoided. The encoding methods must be both sensitive and reproducible. This balance is essential to ensure that the obtained codes retain the complexity of the original patterns while retaining consistent response to the challenge under different measurement conditions (i.e., a low variance value). The robustness of the PUF can be further quantified by the bit stability, representing the ability to produce reliable responses in various operating conditions. Recent efforts in the field tend to introduce pattern‐matching algorithms for smart authentication approaches. Their core mechanisms are to extract features from the PUF response and give verification output through pattern similarity measurement and threshold determination.^[^ ^182^ ^]^ These algorithms typically have low computational complexity, resulting in fast processing speed and good scalability.^[^ ^183^ ^]^ In this section, we will discuss examples about the generation of PUF devices based on different optical probes. Some selected examples of PUF devices, their constitutive materials, performance metrics, stability features, and National Institute of Standards and Technology (NIST) validation status are summarised in **Table** **1**.

**Table 1:** Key features and encoding capacity of recently reported PUFs.

Materials	Fabrication process	Type of readout	Encoding capacity	NIST tests	Bit uniformity	Uniqueness ^c)^	Repeatability/Stability	Refs.
Organic dye	Spin coating Thermal annealing	Fluorescence, Lifetime, Raman, IR	n.d.	n.d.	0.503	0.4901	>90% (100 readouts) UV (365 nm, 3 W, 1 h)	[184]
Organic dye nanolaser arrays	Ion etching Asymmetric liquid breaking	Laser emission	2^2 048^ ^a)^	Passed	n.d.	0.49	Thermal (70 °C, 1 h) Error bit <0.5%	[185]
Phosphorescent organic crystals	Drop casting on MoS_2_ films	Confocal microscopy, CCD camera	2.74 × 10^17^ (1 × 1 group, Np6A/Np6) ^a)^	n.d.	n.d.	0.3316 (1 × 1 group, Np6A/Np6)	>98% (1 × 1 group, Np6A/Np6)	[190]
Chiral liquid crystals	Capillary force injection	Polarised optical microscopy	10^55^ ^b)^	n.d.	0.5007	0.5020	Intra‐HD = 0.0081 Thermal (100 °C)	[191]
DR1‐doped Liquid crystal droplets	Emulsion Thermal polymerization Curing	Scattered light (speckle pattern)	2^1 750^ ^b)^	n.d.	n.d.	≈ 0.50	Repeated use (1000)	[192]
CD‐doped nanofibers	Electrospinning	Dark‐field scattering, RGB fluorescence	5^246^ ^b)^	Passed	0.49341	0.4908	Intra‐HD = 0.08146 Thermal Humidity, irradiation	[198]
Monosaccharide CDs	Nanoprinting‐assisted flash synthesis	Fluorescence, White‐light interferometry	10^63 593^ ^a)^	n.d.	0.492	0.498	>93% (fluorescence) 87% (interferometry)	[199]
SPNs	Photolithography	Confocal laser scanning microscopy	10^220 322^ per mm^2^ ^a)^	n.d.	0.4948	0.5000	Intra‐HD = 0.05568 UV (365 nm, 24 h) Thermal (‐20 °C to 40 °C) Humidity (85%)	[203]
Lanthanide‐doped zeolites	Mixing in PVA solution Spin‐coating on glass	Excitation‐dependent fluorescence	6 × 10^104^ ^b)^	n.d.	n.d.	n.d.	18 months (ambient conditions) Water‐sensitive	[207]
RE^3+^‐doped silica nanocomposites	Bionic soft replication	Confocal laser scanning microscopy	10^10 000^ ^a)^	n.d.	0.4994	0.7709 (decimal key)	Thermal (up to88% stability, 30 × 1000 °C)	[208]
Anisotropic RE flakes	Chemical vapor deposition	Polarised luminescence	2^380 000^ ^a)^	n.d.	≈ 50%	≈ 50%	Intra‐HD ≈ 5%	[212]
MoS₂/TiO₂ heterostructures	Chemical vapor deposition Drop casting	Photoluminescence mapping	3.55 × 10^131^ ^b)^	Passed	50%	0.5045	Over 30 days (> 96% correlation)	[217]
II‐VI QDs	Spin coating Inkjet printing	Fluorescence, Smartphone microscope	4.7 × 10^202^ per pattern ^b)^	n.d.	n.d.	n.d.	Chemical and photo stability (over 2 months)	[218]
CsPbBr₃ perovskite NC films	Laser engraving Evaporative self‐assembly	Fluorescence, Smartphone authentication	2.1 × 10^623^ ^b)^	n.d.	n.d.	n.d.	Humidity sensitive (unprotected) Stable for >60 days (PDMS‐protected)	[225]
Metal halide perovskite NPs (MAPbBr_3_)	In‐situ photosynthesis Spinodal polymer decomposition	Fluorescence	10^243 834^ per mm^2^ ^a)^	n.d.	≈ 0.4977 (low‐level PUF)	0.5033 (low level PUF)	>9 months (ambient conditions)	[227]
Cs₂AgBiBr_6_ PQD	Coating with polymer blend	Fluorescence	2^98 888^ ^a)^	n.d.	0.5	0.5004	Intra‐HD = 0.0396 87% stability after 90 days (35 °C)	[229]
AuNPs on P2VP	Spin coating of polymer films Thermal annealing Drop‐casting of AuNPs	Light reflection, SERS	n.d.	n.d.	0.5352	0.4695	Intra‐HD ≈ 0 Stable for >1 year (ambient conditions)	[232]
Mie‐resonant silicon NPs	LIFT	Dark field microscopy, Raman	10^240 000^ (1000 × 500 pixels) ^a)^	n.d.	n.d.	n.d.	Stable for >1 year (ambient conditions)	[235]
GERTs	Drop casting	Raman	3 × 10^15 051^ (50 × 50 pixels) ^a)^	n.d.	n.d.	n.d.	94% for binary encoding 84% for quaternary encoding	[238]
Dye‐loaded Au‐coated Si nanorods	Drop casting	Fluorescence, Raman, Smartphone authentication	6.43 × 10^24 082^ ^a)^	n.d.	n.d.	n.d.	Repeatability ≈98% after 1000 cycles	[241]
Cellulose nanocrystal, PVP‐K30, glycerol	Evaporation‐induced self‐assembly	Structural color, Hyperspectral imaging	2^2 304^ ^a)^ (reflectance) 2^22 500^ ^a)^ (optical image)	Passed	0.4964	0.4912	Repeatability ≈ 91% after 20 cycles of 80 °C at 99% humidity	[246]
Polymer photonic crystal hydrogels	Electrostatic self‐assembly Photopolymerisation	Optical microscope (structural color)	2 × 10^166 055^ ^a)^	n.d.	n.d.	n.d.	Mechanical (stretchable)	[247]
PMMA microspheres on K9 glass	Spin coating Electron‐beam evaporation	Total internal reflection, Smartphone authentication	2^370^ ^b)^	Passed	0.498	0.500	Intra‐HD = 0.068 Mechanical (30 min ultrasound) Thermal (90 °C, 24 h) Chemical (brine, NaHCO_3_, AcOH)	[250]
Diamond microparticles	Chemical vapor deposition	Fluorescence, Dark field microscopy	n.d.	n.d.	0.4996	0.5000	Chemical (1 M NaOH, 1 M HCl, brine) Thermal (400 °C, 24 h) Light (365 nm, 2 h; 532 nm, 2 h)	[252]
Silk fibroin‐embedded microdiamonds	Solution mixing Spin coating Water bath annealing	Raman	2^10 000^ ^a)^	n.d.	0.4997	≈ 0.50	Photo‐stable Stable to corrosion Biocompatible	[260]
Silk fibers	Native silk cocoons (optical material) 3D printing (device)	Lens‐free fluorescence microscopy	2^768^ ^a)^ 2^345^ ^b)^	Passed	0.4972	0.4990	Intra‐HD = 0.0309 Flexible device Thermal (65 °C) High humidity (6 days): error rate of 0.0004	[196]

^a)^ Maximum theoretical encoding capacity;

^b)^ Effective encoding capacity;

^c)^ Uniqueness given as the inter‐device Hamming Distance. n.d. denotes “no data available”.

### 3.1 PUF Devices Based on Organic Inks

#### 3.1.1 PUFs From Organic Dyes

**Figure 7:**
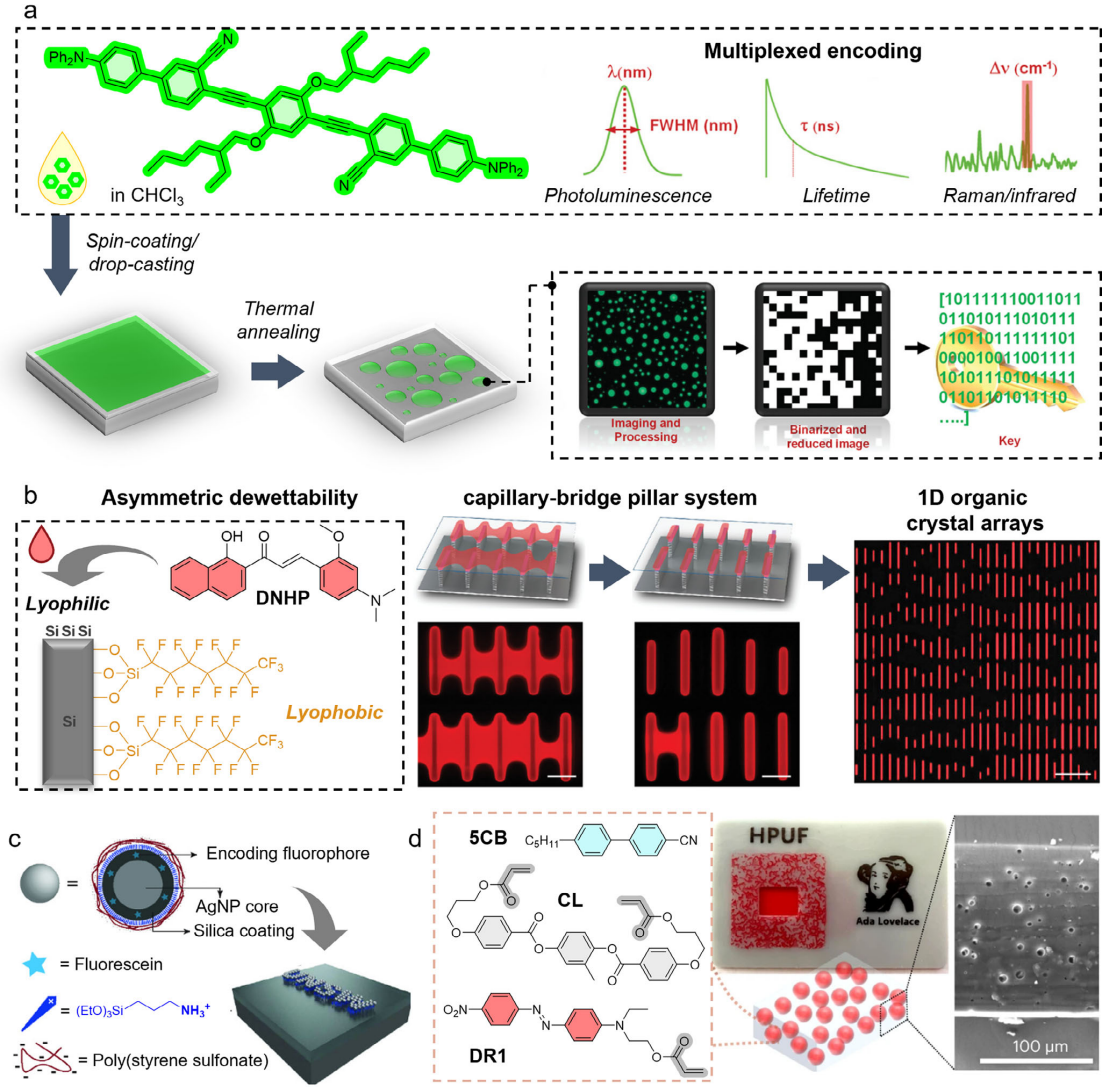
PUF devices obtained by processing organic dyes. a) An organic light‐emitting PUF device with green emission was spin‐coated and annealed to form random microsphere patterns. The resulting fluorescent images were digitized into security keys. Additional security layers could be added thanks to the unique properties of the dye. Reproduced with permission^[^ 184] Copyright 2021, John Wiley and Sons. b) Asymmetric liquid breaking was driven by the asymmetric dewettability of lyophilic/lyophobic silicon nanopillars, resulting in random 1D organic crystal nanolaser arrays of DNHP. Reproduced with permission^[^ 185] Copyright 2019, John Wiley and Sons. c) Electrostatic effects between positively charged substrate and negatively charged NP probes formed unclonable micropatterns. Reproduced with permission^[^ 186] Copyright 2016, John Wiley and Sons. d) Disordered photonic media composed of LC droplets (composition shown on the left) were manufactured into a PUF device. A picture of the PUF inserted in a 3D‐printed authentication card is shown (middle). A scanning electron microscopy image of the side view of the film is reported on the right. Reproduced with permission^[^ 192] Copyright 2024, The Author(s), under exclusive license to Springer Nature Limited.

PUF devices based on organic fluorophores can be designed by randomly distributing taggants during film‐forming approaches. Onses et al. spin‐coated a green‐emissive hot‐exciton oligo(*p*‐phenyleneethynylene) derivative to form a nanofilm (**Figure** **7**a).^[^ ^184^ ^]^ Followed by a brief thermal annealing at a modest temperature (170 °C), the nanofilm dewetted to form randomly positioned/sized hemispherical features with bright fluorescence. The fluorescence images were then captured and binarised with a reduced size to generate security keys. The security keys digitized from the fluorescent hemispherical features exhibit high uniformity (0.50) and uniqueness (0.49), proving the successful design of a PUF device with organic fluorophores (Table 1). Meanwhile, the unique photophysical and structural properties of the dye (i.e., fluorescence profile, excited‐state decay dynamics, Raman mapping/spectrum, and infrared spectrum) formed the basis for the additional security layers.

Beyond straightforward film‐forming methods, PUF devices can be made by modifying substrates^[^ ^185^ ^]^ or probes^[^ ^186^ ^]^ with organic dyes. Flexible PUF tags were prepared by synergistic combination of electrospinning and electrospraying using PVA doped with fluorophores S420 and rhodamine B).^[^ ^187^ ^]^ Electrosprayed microdroplets were deposited onto a dense layer of nanofibers, thereby generating random patterns with three encryption levels based on droplet mapping, fluorescent tag spectra, and speckle patterns. The Wu group fabricated a micropillar nanolaser array by photolithography and deep reactive‐ion etching.^[^ ^185^ ^]^ They then treated the sidewalls and bottoms of the micropillars with heptadecafluorodecyltrimethoxysilane solutions to form a lyophobic surface, while the top of the micropillars remained lyophilic. When a low volume of solution with fluorescent dye (*E*)‐3‐(4‐(dimethyamino)‐2‐methoxyphenyl)‐1‐(1‐hydroxynaphthalen‐2‐yl)prop‐2‐en‐1‐one (DMHP) was placed on the surface of the micropillar arrays, the modified structure yielded asymmetric liquid breaking instead of a symmetric splitting of the liquid film (Figure 7b). With the evaporation of the solvent, unequal quantities of organic molecules in each discrete capillary bridge yielded organic crystals with random lengths. The resulting device successfully passed the statistical standardization tests of the NIST (Table 1). Liu et al. synthesized fluorescent organosilica nanodots by hydrothermal treatment of organosilanes and rhodamine B.^[^ ^188^ ^]^ During the dewetting process, the nanodots formed nano‐island structures with random disordered arrangements, thus providing unclonable features for the device. Gooding et al. modified both the substrates and the security ink NPs to drive the design of PUF devices (Figure 7c).^[^ ^186^ ^]^ The electrostatic effect was the fundamental force to promote the self‐assembly of the NPs, which later formed unclonable plasmonic security labels. Specifically, the silica substrate was treated with 3‐aminopropyltriethoxysilane (APTES) solution to form a positively charged surface. The NPs were subsequently coated with an inner layer of APTES and an outer layer of negatively charged poly(sodium 4‐styrenesulfonate). As a result of electrostatic adsorption between substrate and NPs and electrostatic repulsion between the NPs, unclonable micropatterns were formed by the random arrangement of discrete probes. By further incorporating shadow mask lithography to the fabrication process, the modified probes were able to be conveniently embedded into a variety of 2D patterns.

Moreover, an interesting strategy relying on fluorescent blinking was reported by Lu et al.^[^ ^189^ ^]^ The authors embedded organic fluorophores (rhodamine B, fluorescein, *p*‐COOH‐RB‐C2, and Cy5) into Ag@SiO_2_ core–shell nanocubes. These embedded blinking dyes were drop‐deposited on a micropatterned Ag film which randomly formed nanoclusters. The encryption of the PUF then relies on the “on” and “off” states of the blinking, corresponding to the binary bits “1” and “0.”

In optically active organic crystals, the intrinsic randomness of the crystallization process has been used to prepare PUF devices. Drop‐casting 2,5‐dihydroxyterephthalate (DDT), 5‐bromo‐2,6‐dihexyloxy‐1‐naphthaldehyde (Np6A) and 1,5‐dibromo‐2,6‐dihexyloxynapthalene (Np6) onto a pre‐patterned MoS_2_ substrate generated organic crystals with green and red chaotic phosphorescent patterns.^[^ ^190^ ^]^ Each DDT and Np6A/Np6 PUF domain was reported with a producible encoding capacity of 6.11 × 10^6^ and 2.74 × 10^17^ respectively (Table 1). Furthermore, chiral LCs were also used for PUF devices due to their polarisation‐dependent optical properties.^[^ ^191^ ^]^ A nearly random maze of LCs was generated by spontaneous orientational symmetry breaking. The two different geometries in the adjacent air cavities of the mazes were digitized into binary codes by a pattern recognition algorithm. The actual encoding capacity of the device was moderate, but the uniformity, uniqueness, and repeatability were close to ideal (Table 1). An all‐optical multilevel PUF was also prepared by Nocentini et al. using PDMS films containing randomly dispersed dye‐doped LC droplets (Figure 7d).^[^ ^192^ ^]^ In this work, the encoding was based on reversible changes in scattering properties occurring in **5CB** LC droplets upon irradiation with blue light. This was promoted by loading the Disperse Red 1 (**DR1**) azobenzene dye inside the droplets to generate a photothermal effect. A bis‐acrylate cross‐linker (**CL**) was also added to the ink formulation to generate a stable polymeric network facilitating the LC alignment recovery after phase transition. The PUF was manufactured by dispersion of LC emulsion droplets within the polymer matrix, followed by thermal polymerization, which created complex, unclonable random patterns. This yielded a reconfigurable PUF with deterministic, stable, and repeatable transformation between two phases that was interrogated with red light. The encoding capacity of these PUFs was significantly enhanced by the multilevel operation, with the potential to generate keys with up to 1750 independent bits (Table 1).

An elegant strategy described by Xu et al. combined fluorescent probes with holographic encryption to prepare low‐cost angle‐dependant multiplexed PUF devices.^[^ ^193^ ^]^ The system encrypts information as computer‐generated holograms and uses an improved Diffie–Hellman key exchange for secure parameter transfer, offering robust, low‐cost, and misjudgement‐free encryption. Finally, pyrene‐based AIEgens developed by Chen et al. were used in the preparation of a PUF device with water‐responsive features.^[^ ^194^ ^]^ When exposed to water infiltration, the incorporation of AIEgen side‐chain domains within a poly(*N*‐isopropyl acrylamide) copolymer led to PL switch‐on due to polymer shrinkage resulting in unique, non‐replicable AIE patterns. Following up on this work, the same group recently reported the preparation of an AIE PUF device using a similar pyrene dye.^[^ ^195^ ^]^ Randomness was introduced during the dewetting process, and controlling the packing of the aggregates could lead to dual‐wavelength emission properties.

#### 3.1.2 PUFs From Organic Conjugated Nanomaterials

**Figure 8:**
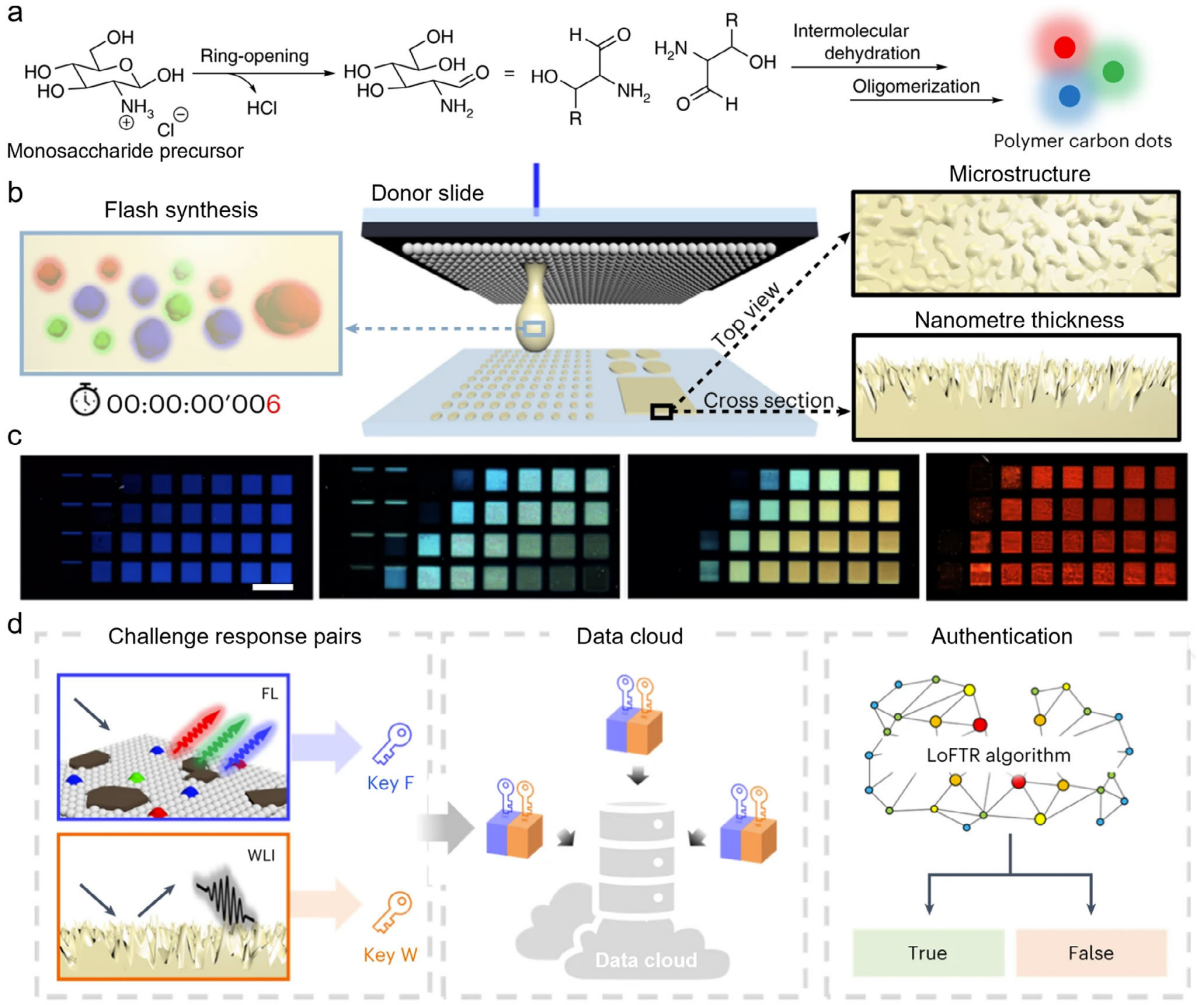
CD‐PUF device synthesized by laser‐assisted flash process. a) Proposed reaction process of polymer CDs. b) The synthesis and patterning of CDs were achieved simultaneously within milliseconds. c) Selected nanofilms from the experimental library. d) Workflow for PUF key generation, database initialization and authentication. Reproduced under the terms of the CC BY license [199] Copyright 2023, The Authors. Published by Springer Nature Limited.

In recent several years, random nanofibers generated from natural sources^[^ ^196^ ^]^ or by electrospinning^[^ ^197^ ^]^ have rapidly become candidates for PUFs. Ding et al. embedded multicolor CDs in a mixed polymer matrix to label individual fibers, generating four independent CRPs per PUF: dark‐field scattering, fluorescent red, green and blue emissions.^[^ ^198^ ^]^ Then, they used perceptual hashing to extract the frequency features of random nanofiber patterns and digitise them into a fingerprint (Table 1). Embedding CDs in these stochastic processes can be straightforward, however, it requires pre‐synthesis of the CD nanoparticles and often a post‐treatment step to avoid ACQ. One of the present authors was involved in a recent report of a CD‐based PUF device that simultaneously synthesized CDs with tuneable emissions and printed nanofilms with random patterns.^[^ ^199^ ^]^ Environment‐friendly precursors, monosaccharides, underwent a ring‐opening reaction, followed by intermolecular dehydration and oligomerization upon thermal treatment to generate polymer CDs (**Figure** **8**a). These processes were achieved by flash synthesis in a continuous laser‐based printing system (Figure 8b). The precursors were prepared as a thin film on a so‐called “donor” slide. On top of the precursor layer, an absorber layer was added to exploit the heat of the laser irradiation heat. The temperature of the absorber layer could go above 500 °C within milliseconds of irradiation, therefore supporting the thermal conditions for the synthesis of CDs with different emissions (Figure 8c). With the obtained CD‐PUF devices, fluorescent and thickness patterns were both digitized as keys (Table 1), and stored on a data cloud. An open‐source feature‐matching algorithm was further introduced for facile identification by end‐users (Figure 8d). As an added benefit from the ultrafast synthesis process, a nanofilm library (1920 experimental datasets) with tuneable solid‐state fluorescence from violet‐blue to red and different microstructures was generated. Therefore, besides offering an advanced PUF device, this technique could be useful for future synthesis of color conversion layers, quenching‐resistant (organic) light‐emitting diodes, or sensitive bio‐/chemosensors. In another example of conjugated carbon nanomaterials, stiff photo‐crosslinkable polymers were spin‐coated onto an elastic carbon nanotube and PDMS layer to generate dynamic wrinkles. Infrared irradiation of the bilayer erased the pattern via cycloaddition of anthracene‐containing side‐chains, and the surface reversed to its original fingerprint pattern at room temperature with preserved surface memory, offering a facile and robust strategy for responsive PUF devices.^[^ ^200^ ^]^

**Figure 9:**
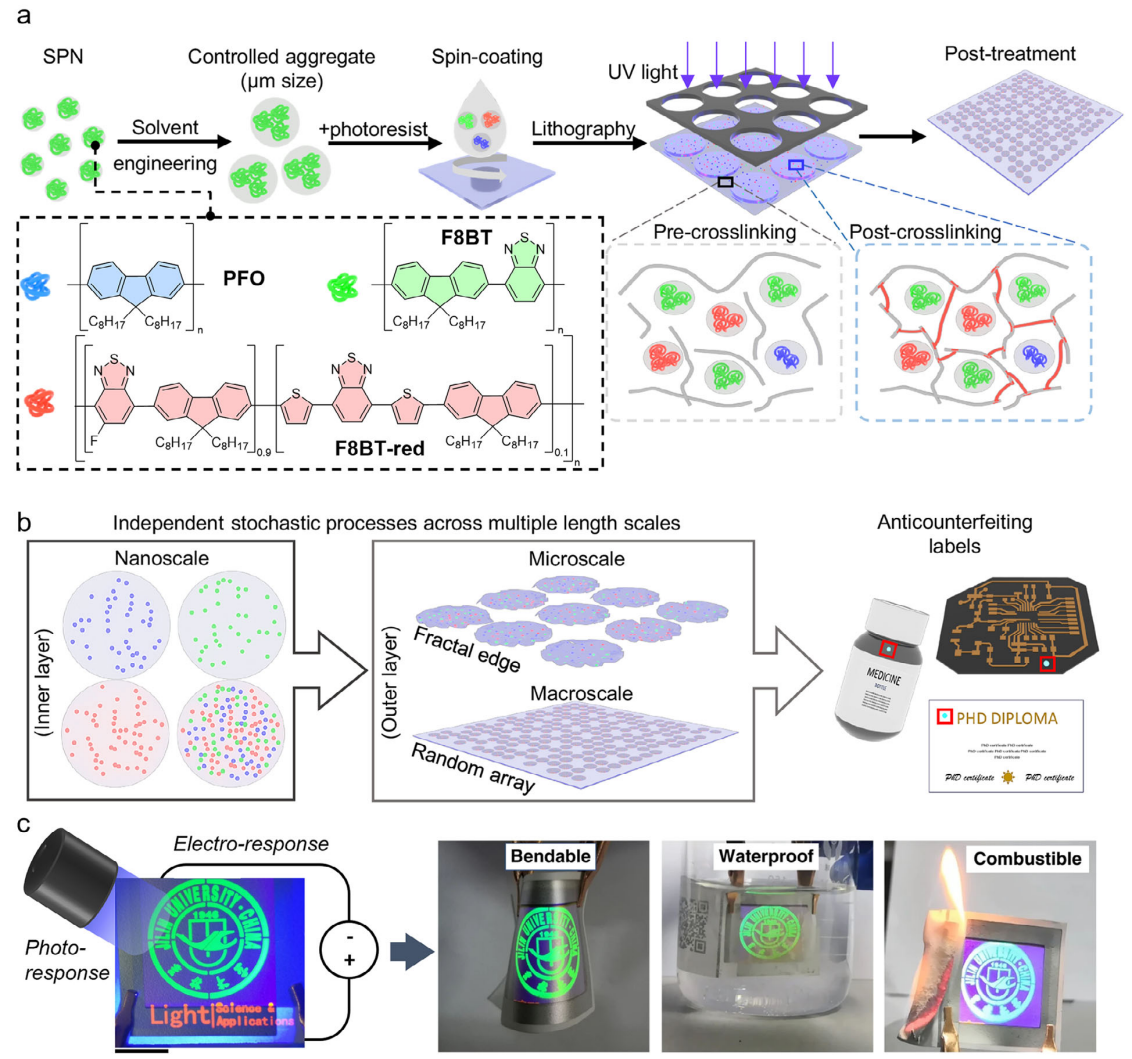
PUF devices prepared from light‐emitting polymers. a) Semiconducting polymer NPs prepared by nanoprecipitation of polymers emitting RGB colors under the same excitation wavelength, were mixed with photoresists and treated by a photolithography process to prepare PUF devices. b) PUFs were encoded at the nano‐micro‐ and macroscale thanks to the random distribution of SPNs in each microspot. Fractal edges and partially removed arrays were generated during photolithography. The PUF devices were applied as anticounterfeiting labels for different products. Reproduced under the terms of the CC BY license [203] Copyright 2025, The Authors. Published by Springer Nature Limited. c) An organic light‐emitting device, encoding different information based on light and voltage exposure, exhibited highly desirable mechanical, water, and fire resistance. Reproduced under the terms of the CC BY license [204] Copyright 2022, The Authors. Published by Springer Nature Limited.

Semiconducting polymers stand for another important type of conjugated organic nanomaterials that have been used as processable optical materials for PUFs. Highly crystalline carbon nitride (HCCN) is an organic semiconductor polymer with the capacity to store electrons upon light irradiation. This is believed to be the source of a reversible, “photochromic‐type” color change that was exploited in PUF films.^[^ ^201^ ^]^ A photoswitchable color‐changing PUF was for example generated by stochastic distribution of HCCN/cellulose nanofibers in poly(vinyl acetate) (PVAc).^[^ ^202^ ^]^ Our group also developed highly secure PUF devices using semiconducting polymer nanoparticles (SPNs) embedded in photoresists.^[^ ^203^ ^]^ SPNs were prepared from three CPs, i.e., blue emissive poly(9,9‐di‐n‐octylfluorenyl‐2,7‐diyl) (PFO), green‐emissive poly(9,9‐dioctylfluorene‐*alt*‐benzothiadiazole) (F8BT) and red‐emissive poly[(9,9‐dihexylfluorene)‐*co*‐2,1,3‐benzothiadiazole‐*co*‐4,7‐di(thiophen‐2‐yl)‐2,1,3‐benzothiadiazole] (F8BT‐red), thereby allowing RGB encoding while exhibiting high brightness, photostability, and size tuneability compared to other fluorescent taggants such as QDs and CDs (**Figure** **9**a). Large, easily detectable SPNs were embedded in polymer matrices and photoresists using spin coating and photolithography techniques, resulting in patterns that could withstand harsh conditions, such as immersion in artificial sweat. The PUFs were designed with three levels of stochastic encoding at the nanoscale, microscale, and macroscale features. The random distribution of SPNs within microspots, fractal edges on microspots, and random microspot arrays significantly increased the encoding capacity and security of the PUFs (Figure 9b). These features were analyzed using fractal dimension calculations and entropy measurements, confirming their random and unique character (Table 1). A deep‐learning model to achieve precise and fast identification of the PUFs, demonstrating excellent performance in terms of uniqueness, reliability, and bit uniformity. The PUFs were finally tested for practical applications by attaching them to surfaces such as silicon wafers, plastic sheets, aluminum foil, and paper. The devices demonstrated excellent stability under prolonged UV exposure and various storage conditions, maintaining high reliability and low bit error rates. PUF authentication could also be performed using a portable, low‐cost fluorescent microscope, therefore increasing their potential for real‐world applications.

Organic light‐emissive devices (OLEDs) have also been investigated for security applications thanks to their processability and ability to generate both electro‐responsive and photo‐responsive patterns. Multiple stimuli‐responsive anticounterfeiting devices were prepared by dip‐coating paper with a green OLED material, therefore generating unclonable patterns with bendable, waterproof, and fire‐proof characteristics (Figure 9c).^[^ ^204^ ^]^

### 3.2 PUF Devices Based on Inorganic Inks

#### 3.2.1 PUFs From Metal Complexes and Rare‐Earth Materials

**Figure 10:**
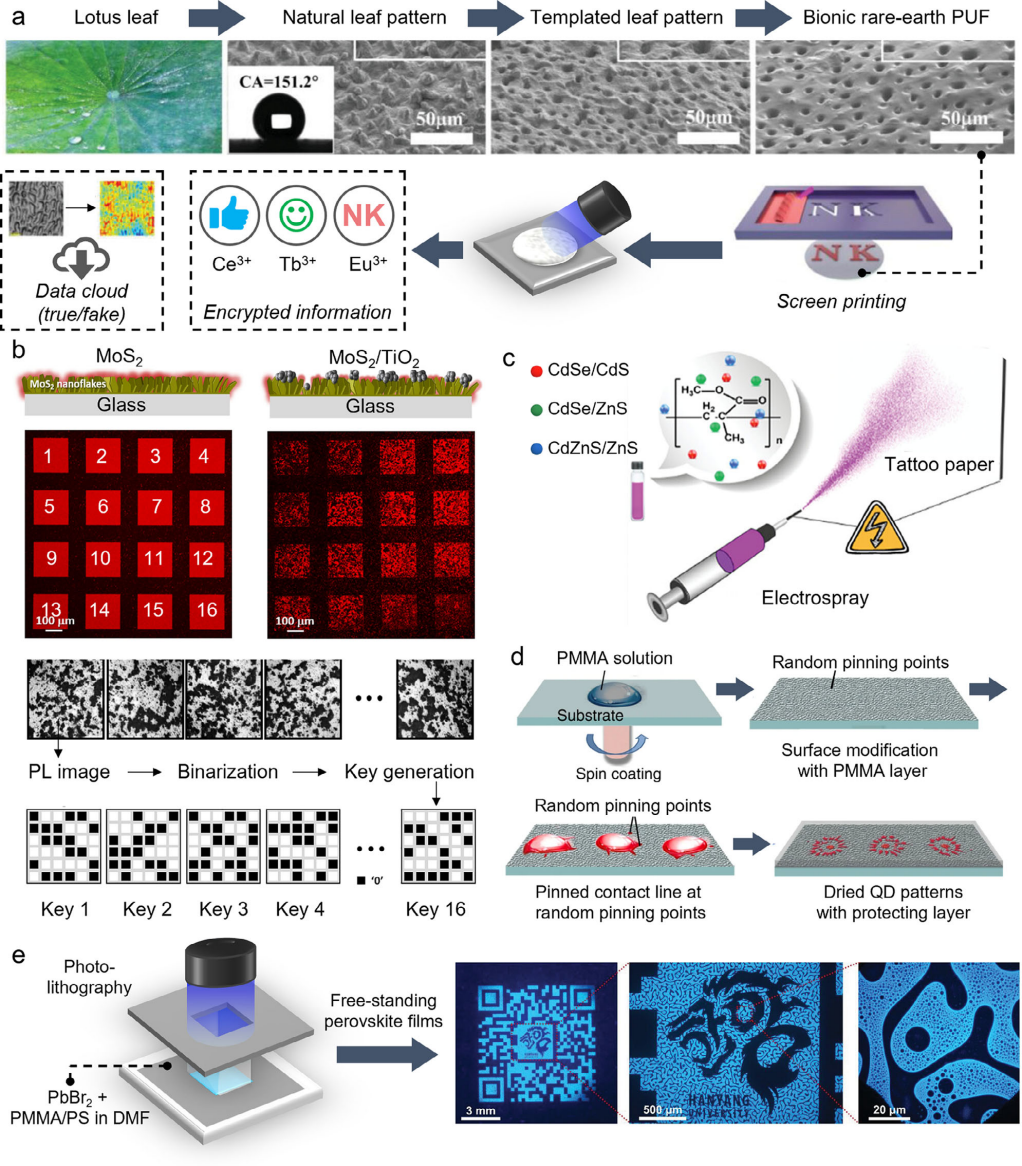
Strategies to fabricate PUF devices with QD inks. a) Nature‐inspired bionic multi‐RE^3+^ PUFs were prepared by soft replication of lotus leaves and screen‐printing luminescent patterns. SEM images of the natural leaves (left), templated leaves (middle), and bionic PUF (right) are shown. The insert shows the contact angle value of the fresh leaves. Reproduced with permission^[^ 208] Copyright 2023, John Wiley and Sons. b) Fluorescent MoS_2_ films were modified by TiO_2_ NPs, which partially quenched emission and formed random micropatterns. Reproduced with permission^[^ 217] Copyright 2021, American Chemical Society. c) Multicolor QD inks were mixed with a PMMA matrix and randomly distributed on a tattoo paper by electrospray. Reproduced with permission^[^ 216] Copyright 2023 The Authors. Published by John Wiley and Sons. d) A substrate was pre‐modified by a PMMA film, offering pinning points to form microdroplets with random edge structures. Reproduced under the terms of the CC BY license [218] Copyright 2019, The Authors. Published by Springer Nature Limited. e) The preparation of MAPbBr_3_‐PMMA/PS composite films by PbBr_2_‐promoted photolysis of DMF leads to phase‐separated security unclonable security patterns. Reproduced with permission^[^ 227] Copyright 2022, John Wiley and Sons.

Luminescent metallic complexes have been used to fabricate PUF devices. Han et al. reported the self‐assembly of a cupric bromide complex of pyrazine 1,4‐dioxide on lithographically defined plasmonic gold surfaces to create PUF devices.^[^ ^205^ ^]^ Interestingly, the use of a thin gold plasmonic underlayer enhanced the color contrast and resolution by coupling to surface plasmons and providing strong resonant scattering. The readouts here included fluorescence and 2D graphical information visualized under bright‐field, dark‐field, and fluorescence microscopy. Another attractive option for PUF devices is the use of lanthanides that facilitate multiplexing and high encoding capacity thanks to their narrow absorption/emission bands.^[^ ^206^ ^]^ A comprehensive anticounterfeiting system containing lanthanide‐based PUF keys, hardware reader, image analysis, and authentication software was developed by Carro–Temboury et al.^[^ ^207^ ^]^ The authors doped zeolites with europium(III), terbium(III), or dysprosium(III) ions, then immobilizing zeolites in a thin PVA film. The film achieves excitation and emission resolved response a maximal encoding capacity up to 7^3600^, and an actual capacity of 6 × 10^104^ for the manufactured device (Table 1). Inspired by the random structure of plant leaves, Yang et al. proposed a PUF system using rare‐earth doped silica nanocomposites soft‐replicating the random but characteristic micropatterns of ginkgo and lotus leaves (**Figure** **10**a), which led to very high encoding capacities (Table 1).^[^ ^208^ ^]^ Multicolor nanorods made from a Y_2_O_3_ lattice and lanthanide ion dopants (Eu^3+^, Tb^3+^, and Ce^3+^) were also manufactured into multi‐color security codes by dispersion into a poly(vinyl chloride) (PVC) matrix.^[^ ^209^ ^]^ Their 2D PL intensity distributions at high resolution showed potential as unclonable codes. Besides, a europium complex bearing coumarin‐sensitizing ligands was manufactured into unclonable cloud‐like patterns by Li et al.^[^ ^210^ ^]^ The powdered complex was directly deposited into a resin before curing the luminescent material randomly at different depths in the device and affected the photoluminescence intensity of the compound to add complexity.

Chiral lanthanide complexes with circularly polarised luminescence have also been proposed as unclonable inks in combination with organic dyes and transparent polymer matrices.^[^ ^211^ ^]^ Anisotropic rare earth Er_3_O_4_Cl flakes, another type of material with polarised luminescence, were synthesized by random chemical vapor deposition onto PDMS to give unique patterns and high theoretical encoding capacity of 2^380000^ (Table 1).^[^ ^212^ ^]^ Downshifting cerium‐activated microphosphors randomly dispersed in a PDMS matrix were also proposed as a smartphone‐readable PUF device.^[^ ^213^ ^]^

#### 3.2.2 PUFs From Inorganic Quantum Dots

To develop a practical PUF, multiple semiconductor nanocrystals must be deposited on a surface in a stochastic process. Different methods have been established to embed QDs and form random patterns. These processes can be categorized in two types of methods: i) QD inks can be randomly distributed by film‐forming methods such as spin‐coating, drop‐casting, etc.^[^ ^214^, ^215^ ^]^ For example, the Onses group reported tattoo‐like multi‐color PUFs by electrospraying a mixture of poly(methyl methacrylate) (PMMA) and red, green, blue‐emitting semiconductor nanocrystals on a temporary tattoo paper (Figure 10c).^[^ ^216^ ^]^ Then the unclonable surface features can be conveniently transferred to a target object using a tattoo‐approach. ii) Pre‐ or post‐modifying the substrate with polymers or NPs to form a rough surface. In this case, even if the QD inks are delivered in a deterministic method, random patterns are still formed by the interaction between the inks and the rough surface. Kim et al. post‐modified MoS_2_ fluorescent films by aggregated TiO_2_ NPs, which suppress the fluorescence via electron transfer (Figure 10b).^[^ ^217^ ^]^ The obtained fluorescent films with random microstructures were then digitized into binary codes for authentication and were in accordance with NIST standards (Table 1). The Qian group generated micro‐droplets with random edges by modifying a substrate with a thin PMMA film (Figure 10d).^[^ ^218^ ^]^ The pinning points randomly distributed on a PMMA film supported the ink droplet to form physically unclonable flower‐like QD patterns upon solvent evaporation, which gave impressive encoding capacities of up to up to 10^202000^ per device (Table 1). Torun et al. generated security labels with both stochastic and deterministic components by pH optimization. They prepared negatively charged QD inks and a positive charged pattern. The adsorption of QDs on the patterns could be tuned by the pH value, resulting in conventional uniform labels or selectively adsorbed labels with intrinsic randomness. QDs coated with 3‐mercaptopropionic were also combined with a pre‐patterned, e‐jet printed, pH‐responsive poly(2‐vinylpyridine) polymer to give pH‐dependant stochastic patterns.^[^ ^219^ ^]^ Surface decorations with gold nanoparticles (AuNPs) generating random hotspots with QDs,^[^ ^220^ ^]^ glass microspheres for multicircular patterns,^[^ ^221^ ^]^ or chaotic metasurfaces^[^ ^222^ ^]^ were also reported for the preparation of PUF devices.

#### 3.2.3 PUFs From Perovskites

Perovskite materials with straightforward synthesis processes and high quantum yields have high potential for optical PUF devices.^[^ ^223^ ^]^ Due to their ionic crystal characteristics, exploiting the random crystallization process and further crystal coalescence (Ostwald ripening) of perovskite materials is a common strategy to use to generate PUF devices. By inkjet printing a CH_3_NH_3_PbBr and PbBr_2_ ink formulation, Liu et al. prepared crystals with randomly distributed nuclei and arbitrary‐grown crystal grains onto a glass surface, which led to unique and evolutive fluorescent patterns.^[^ ^224^ ^]^ Crystallization could be terminated by addition of a PMMA layer on the device. The authors demonstrated that the crystallization process can be further tailored by various ultraviolet ozone treatments on the glass substrates. Also based on random crystallization, Lin et al. introduced a pre‐patterning step by laser engraving to generate irregularity on the substrate and used a self‐developed software for efficient identification.^[^ ^225^ ^]^ A CPB nanocrystalline film was formed in the laser‐engraved lyophilic regions, leading to unique micro‐textures with PUF characteristics. Smartphone‐based fast authentication (12.17 s) and high effective encoding capacity (2.1 × 10 ^623^) were obtained with this four‐layer device (Table 1). Another common strategy is to distribute perovskite QDs on a randomized surface. Chen et al. used ion beam etching to generate chaotic metasurfaces on a sapphire/Al/PMMA substrate in a top‐down approach.^[^ ^222^ ^]^ Drop‐casting of green‐emitting CsPbX_3_ perovskite QDs onto the PMMA metasurface generated fluorescent dots at the optical diffraction limit. Vertically aligned CPB perovskite nanowire arrays were also fabricated by Liu et al. via capillary diffusion of the precursor solution through a pre‐patterned anodized aluminum oxide template.^[^ ^226^ ^]^ The nanowire array was densely arranged with random lengths, showing unclonable lasing mappings under a confocal microfluorescence system. An elegant strategy based on in situ perovskite NP synthesis was reported by Nguyen Minh et al.^[^ ^227^ ^]^ The authors synthesized MAPbBr_3_ by UV photolysis of DMF in presence of a PMMA/PS blend (Figure 10e). During UV irradiation, the MAPbBr_3_ NPs form within the polymer matrix, resulting in random bicontinuous and spot‐like patterns arising from phase separation. This phenomenon was used to prepare QR codes with unclonable patterns with very high encoding capacities per high‐level PUF device, and good uniformity and uniqueness values (Table 1). Dynamic polarisation‐dependant PUFs were also prepared from randomly distributed irregular perovskite microspheres that were vapor‐deposited on silicon.^[^ ^228^ ^]^ Finally, as an example of an anticounterfeiting application, PQD‐based PUFs have been proposed as anticounterfeiting tags for leather goods.^[^ ^229^ ^]^ The random creasing and micro‐/nano‐structure of leather combined with the PL properties of Cs_2_AgBiBr_6_ QDs gave a high encoding (up to 2^98 888^) PUF device with good stability, reliability, and bit uniformity (Table 1).

### 3.3 PUF Devices Based on Raman‐Active Inks

**Figure 11:**
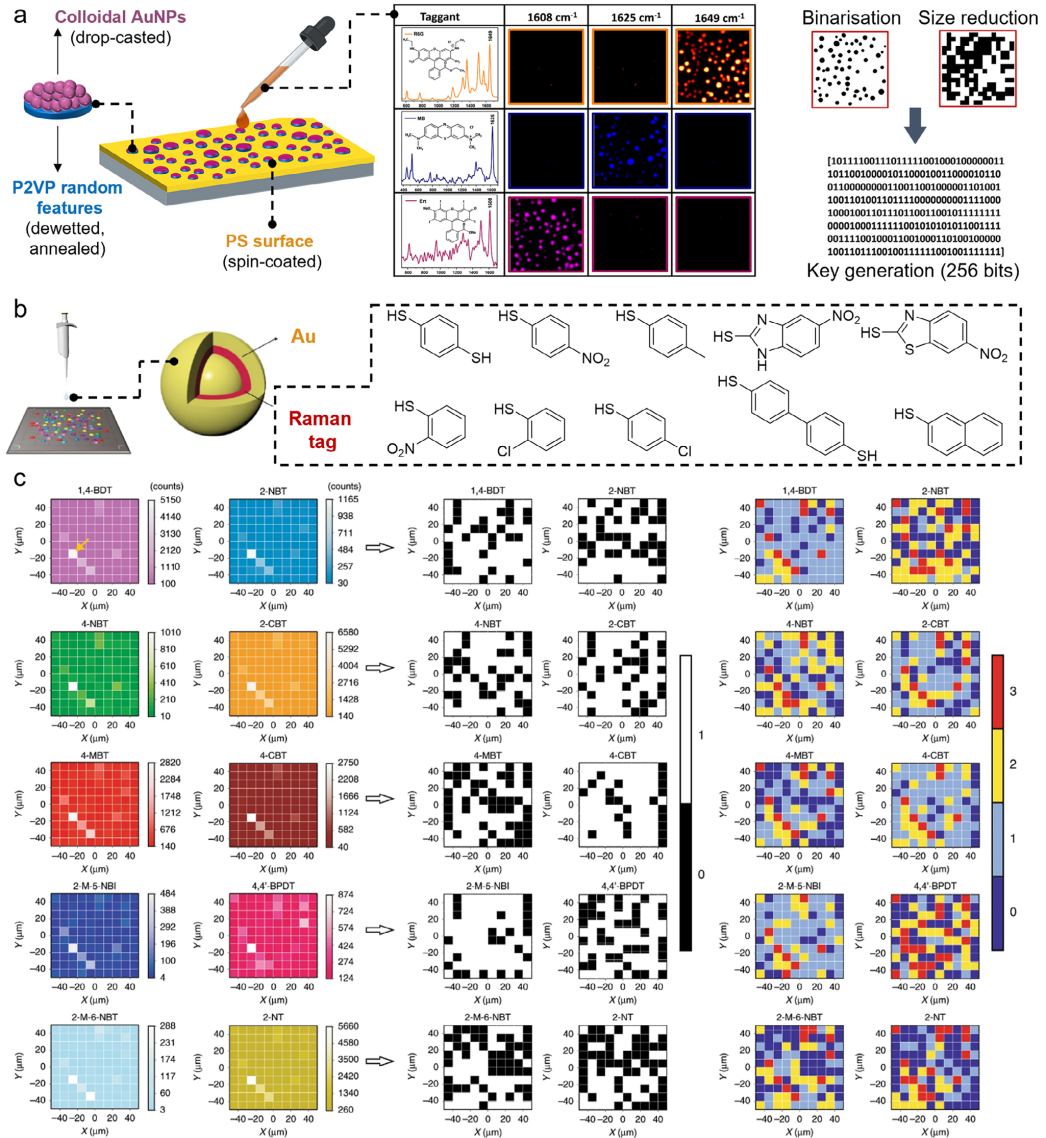
Examples of PUF devices using Raman reporters. a) Random patterns of P2VP over a PS layer were covered with plasmonic AuNPs and Raman tags (Rhodamine 6G, Methylene Blue and Erythrosine) to generate SERS encrypted PUFs. Reproduced with permission^[^ ^232^] Copyright 2021, American Chemical Society. b) A PUF label was generated by drop‐casting GERT NPs constituted of various thiol reporters. c) Plots used for the readout of the PUF label by mapping demultiplexed Raman signals (left), and the corresponding digitizations using binary (middle) and quaternary encoding (right) of Raman intensity levels at each pixel. b) and c) reproduced under the terms of the CC BY license^[^ ^238^]Copyright 2020, The Authors. Published by Springer Nature Limited.

Manufacturing Raman‐based PUFs typically involves processing and functionalizing plasmonic nanomaterials and surfaces in a way that will induce randomness. For example, Tian et al. reported the fabrication of an unclonable device based on the swelling of gelatinous polymer films embedded within plasmonic nanostructures.^[^ ^230^ ^]^ Folding of the films lead to randomized networks containing bright electromagnetic hotspots that significantly increase the Raman signal of embedded Raman reporters. A SERS‐based PUF security label was also fabricated by spin‐coating micelles of diblock copolymer polystyrene‐block‐poly(4‐vinyl pyridine) (PS‐*b*‐P4VP) onto silicon wafers, followed by shadow mask lithography of AuNPs. The resulting patterns were coated with the organic dye malachite green, allowing readout by confocal Raman spectroscopy and the extraction of binary codes for authentication.^[^ ^231^ ^]^ Polymer matrices were also used by Torun et al. who developed a PUF device relying on the inherent randomness of polymer films dewetting. Spin‐coating followed by thermal annealing of poly(2‐vinyl pyridine) (P2VP) on PS‐grafted substrates led to random patterns, that could be read by reflection of visible light and Raman spectroscopy (Table 1) by combination with an additional layer of plasmonic AuNPs (**Figure** **11**a).^[^ ^232^ ^]^ The thermal annealing method was also used to grow noble metal NPs in patterned polymer matrices. Ordered sandwich nanostructures of SiO_2_/Ag/SiO_2_ were annealed after etching the embedded PS sphere arrays, thus creating stable nonreproducible patterns such as hexagonal silver nanoparticle (AgNP) rings, circular AgNP rings, and aggregated AgNPs.^[^ ^233^ ^]^ These unclonable sandwich nano‐patterns were read efficiently using SERS. A universal, fractal‐guided one‐step film annealing strategy was also used by Sun et al. to create random Au networks in a SERS‐based PUF device.^[^ ^234^ ^]^ A dynamic artificial intelligence (AI)‐based authentication system with an expandable database was used by the authors to ensure efficient and reliable authentication with reportedly 0% false positives.

Other technologies, such as LIFT can also be used for the fabrication of Raman‐based PUF devices. Mie‐resonant silicon nanoclusters fabricated by LIFT were reported as size‐dependant Mie‐enhanced Raman reporters, leading to an encoding capacity of up to 10^240000^ for a 1000 × 500 pixel image (Table 1).^[^ ^235^ ^]^ Using a robotic wet‐chemical system, Yu et al. subjected amino silane‐coated AuNPs to in situ epitaxial growth, forming a PUF device.^[^ ^236^ ^]^ The resulting multiscale random features allowed bright field, dark field, and Raman imaging readouts.

Microfluidic techniques can generate nanoprobes for further stochastic nano‐patterning. Gold‐based GERTs from microfluidic fabrication were randomly dispersed in a resin‐based polymer matrix to generate 5D SERS PUFs (reading the frequency and intensity of Raman bands and 3D spaces).^[^ ^237^ ^]^ Drop‐casting of gold‐based GERTs containing ten different thiolated Raman reporters onto silica substrates is also reported to generate PUFs with quaternary encoding of Raman intensities at each pixel over a total of 2500 pixels (Table 1) (Figure 11b,c).^[^ ^238^ ^]^ A formulation of silver nanocubes, functionalized with thiolated Raman reporters, and cellulose nanofibers was used by Cheng et al. to fabricate a multiplexed plasmonic nanopaper by self‐assembly‐assisted vacuum filtration method.^[^ ^239^ ^]^ Fluorescent dyes were also loaded in the cellulose matrix, which allowed localized surface plasmon resonance (LSPR), fluorescence, and Raman encoding. It is worth highlighting that plasmonic materials, that were used for PUF devices with LSPR readouts,^[^ ^240^ ^]^ are generally highly compatible with additional Raman encoding, thus enabling further multiplexing. Multi‐functional hybrid nanoinks with potential for triple‐layer, fluorescent, and plasmonic authentication were also reported.^[^ ^241^ ^]^ The nano‐inks included gold‐coated silicon nanorods with blue emission and three types of fluorescent dyes. Multi‐color fluorescence was then used as a macroscopic smartphone‐compatible readout, complemented by unclonable SERS signals encrypting each pixel at the microscopic level, and shape‐specific aggregated SERS signals at a high level of magnification, leading to a high theoretical encoding capacity (Table 1). Another example with multichannel readout was inspired by the random geometric structures of vascular networks. Kim et al. proposed vein‐like PUFs created by multi‐layer metallic patterns replicated from block‐copolymer (e.g., PS‐*b*‐PMMA) templates.^[^ ^242^ ^]^ Stochastic patterns were created by spin‐casting the copolymers onto a silicon substrate followed by microphase separation. The device was Raman encrypted by coating with plasmonic metal layers, whilst also introducing electrical resistance and optical dichroism readouts.

### 3.4 PUF Devices Based on Other Types of Non‐Resonant Materials

**Figure 12:**
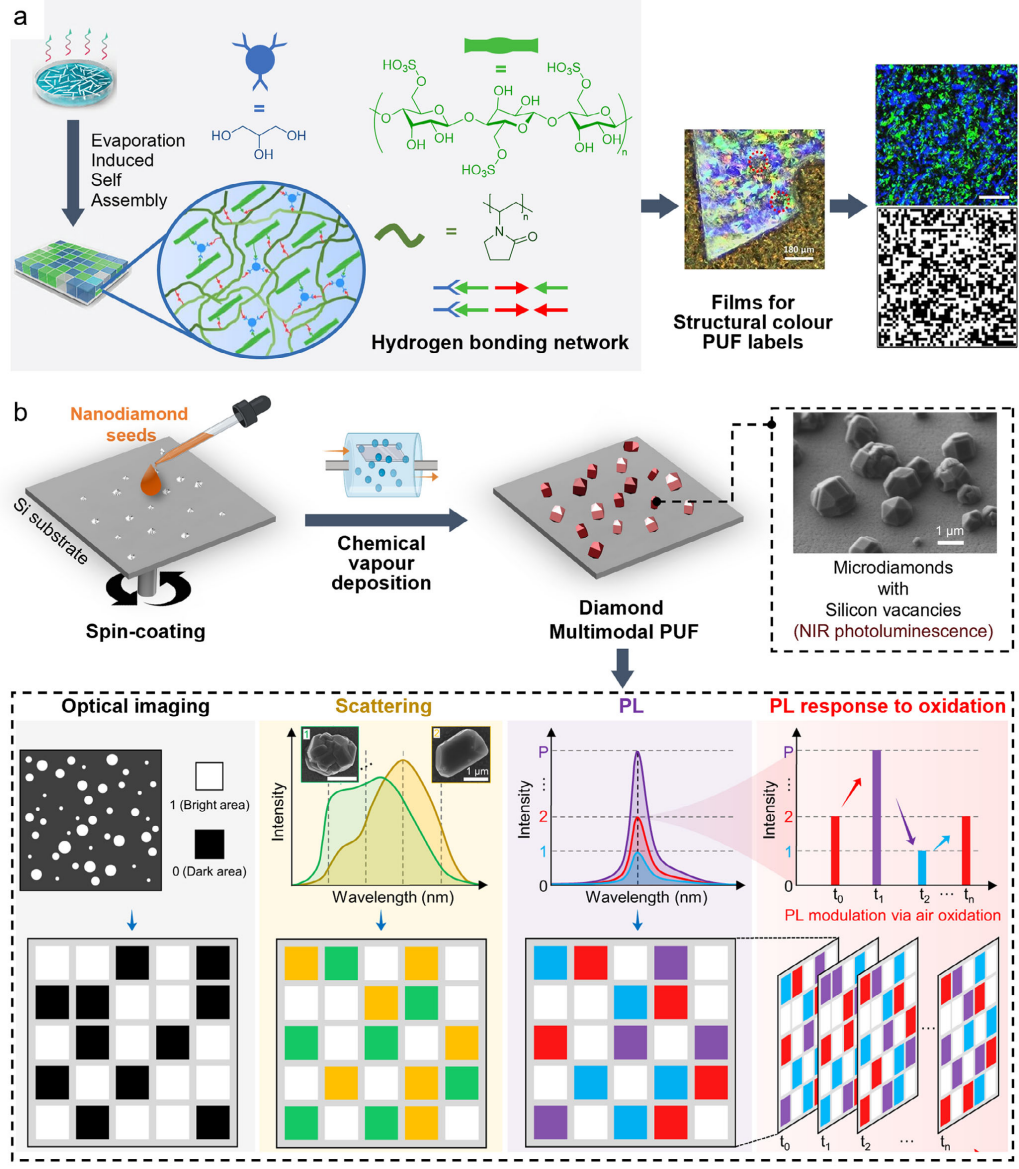
Examples of PUF devices based on reflective or scattering materials. a) Structural color films prepared by evaporation‐induced self‐assembly of hydrogen bond donors and acceptors (cellulose nanocrystals, poly(vinylpyrrolidone), and glycerol) were manufactured into PUF labels. The images on the right show the label and its corresponding 48 × 48 pixels key (scale bar, 500 µm). Reproduced under the terms of the CC BY license [246] Copyright 2024, The Authors. Published by John Wiley and Sons. b) The non‐deterministic fabrication of diamond microparticles from nanodiamond seeds on a silicon substrate led to PUF labels with multiple encoding capacity (bright‐field optical imaging pattern, dark‐field scattering spectrum, PL intensity, and time‐dependent PL modulation via air oxidation for further dynamic encoding). Reproduced under the terms of the CC BY license [252] Copyright 2023, The Authors. Published by Springer Nature Limited.

Structural color is a purely physical phenomenon, produced without any pigment. Therefore, the materials involved typically show long‐term color stability and represent attractive options for the design of optical PUF devices. A large choice of colloidal and nanostructured materials and compatible techniques can be used for the preparation of structural color PUFs, as recently reviewed by Lin et al.^[^ ^243^ ^]^ A straightforward structural color PUF device was prepared with colloidal crystals prepared from injection‐casted poly(styrene‐acrylamide) copolymer nanospheres.^[^ ^244^ ^]^ The resulting PUF exhibited microscopically ordered yet macroscopically disordered stripe patterns. The water‐responsive polymer film allowed the PUF to switch between visible and invisible states, thereby enhancing security. The authors reported a theoretical encoding capability of 10^2500^ for a mapping resolution of 50 × 50 pixels with this device. In another nanosphere‐based example, an unclonable device based on structural color was fabricated using arrays of monodisperse poly(styrene‐methylmethacrylate‐acrylic acid) (P(St‐MMA‐AA)) nanospheres, copolymerized with *N*‐hydroxyethyl acrylamide (N‐HEAA) and inkjet‐printed into computer‐designed patterns.^[^ ^245^ ^]^ Random structural color domains were also prepared by growing tactoids of sulfonated cellulose nanocrystals, poly(vinylpyrrolidone), and glycerol (**Figure** **12**a).^[^ ^246^ ^]^ Random distribution of colors, shapes, sizes, and reflectance spectra was obtained by evaporation‐induced self‐assembly and fusion of tactoids, leading to structural color films that were manufactured into PUF devices with a coding capacity of at least 2^2304^ (Table 1). With their capacity to confine photons in a limited space, photonic crystals have been proposed as another type of material for structural color PUF. Inspired by the random presence of microcrystalline domains in precious opals, Wu et al. developed photonic crystal hydrogels with randomly distributed sparkling spots using a polyacrylamide film with randomly distributed polystyrene‐maleic acid (PSMA) colloidal crystals,^[^ ^247^ ^]^ providing a theoretical encoding of 166 050 possible RGB colors (Table 1). In another example, monodisperse PS NPs inkjet‐printed onto hydrophobic substrates spontaneously assembled into microdomes leading to photonic crystals with structural colors.^[^ ^248^ ^]^ The printed patterns were enriched with spectral absorbers and fluorophores, which generated unclonable photoluminescent speckles that were further analyzed with deep learning algorithms. Plasmonic materials were also used to prepare a hybrid camouflaged PUF integrated onto a paper substrate.^[^ ^249^ ^]^ Generating a multi‐layer optical nano‐cavity composed of thin Ag and ZnO layers on the substrate by sputtering deposition enabled multi‐level authentication via the generation of QR codes, plasmonic structural colors, and of random speckle patterns.

Light reflection was also investigated as a readout for PUF devices. Total internal reflection of light by PMMA microspheres randomly dispersed onto a glass/alumina surface^[^ ^250^ ^]^ reportedly led to a PUF with an encoding capacity of 2^370^ (Table 1). Arenas et al. used cholesteric spherical reflectors made from LCs molded into solid spheres to produce PUF devices.^[^ ^176^ ^]^ These produce unique optical patterns under various illumination conditions that could be captured and analyzed using image subtraction and blob extraction algorithms for reliable and secure authentication. Scattered light, detected and analyzed with the help of computational techniques, can also be used as a readout for PUFs. This was exemplified by Fernández‐Benito et al. who used recyclable polyester NPs as a scattering agent for neural network‐based smartphone authentication.^[^ ^251^ ^]^

Naturally‐occurring or nature‐inspired materials have proven to be an important source of inspiration in the development of novel PUF devices. Diamonds, which are intrinsically robust and unclonable materials, are one example of this. Unclonable labels were produced by exploiting the light scattering properties of microdiamonds, grown onto photoluminescent silicon‐vacancy‐containing substrates by chemical vapor deposition (Figure 12b).^[^ ^252^ ^]^ This combination of robust scattering and luminescent materials allowed multiplexed, time‐dependant encoding with high capacity, excellent uniformity and stability (Table 1). Chemical‐vapor deposited diamond was also used as an attractive unclonable Raman tag (1332 cm^−1^).^[^ ^253^ ^]^ The Raman information was reportedly stored in a 6D space based on color space (RGB filters), greyscale, irradiation wavelength, and power. Microdiamonds with nitrogen‐vacancy color centers embedded in PDMS were used by Guo et al. to manufacture PUF devices with multilevel encoding based on Raman spectroscopy, PL, and optical probe magnetic resonance.^[^ ^254^ ^]^ Interestingly, optical rotation and polarised optical microscopy were also exploited by Park et al. as a PUF readout.^[^ ^255^ ^]^ To achieve this, the authors racemized photonic crystals using spontaneous mirror symmetry breaking of achiral bent‐shaped azobenzene dimers. Phases were controlled by temperature increase, forming racemic helical nanofilaments upon cooling. Besides these non‐resonant detection methods, non‐linear optical materials have also been proposed as PUF devices.^[^ ^256^, ^257^ ^]^

The challenge of product traceability in the food industry was addressed using PUF devices manufactured from highly biocompatible materials such as modified corn starch,^[^ ^258^ ^]^ and silk fibers.^[^ ^259^ ^]^ The latter were for example reported as a support matrix for edible counterfeiting tags in combinations with fluorescent proteins for optical readout. Silk was also used as biocompatible matrix in microdiamond‐based Raman security tags that were implanted under chicken skin for traceability.^[^ ^260^ ^]^ Random distribution was ensured via spin‐coating and water bath annealing, leading to a maximum encoding capability of 2^10000^ for this device (Table 1). Biocompatible and biodegradable silk microparticles were also used as Raman‐readable cryptographic PUF tags that could be drop‐casted onto seeds.^[^ ^261^ ^]^ A sophisticated silk protein‐based microlaser system, fabricated by soft‐lithography casting a laser dye‐doped silk fibroin solutions onto a PDMS template, was also reported.^[^ ^262^ ^]^ Interestingly, randomly distributed fibers of native silk also possess intriguing intrinsic optical properties. They are able to generate spatially chaotic light diffraction, thereby forming self‐focused, high‐contrast spots of light transmitted through the material, which was exploited in PUF devices with near‐ideal encoding properties (Table 1).^[^ ^196^ ^]^ Silk protein films were also used as nucleation regulators in the growth of calcite crystals.^[^ ^263^ ^]^ These random crystals co‐incorporated biocompatible fluorescent dyes to enable multi‐level and eco‐friendly authentication. Additional nature‐inspired or biocompatible systems were reported, including “bionic” scattering films molded from plant leaves that can generate optical speckles.^[^ ^264^ ^]^

## 4 Summary and Outlook

Unclonable anticounterfeiting devices have the potential to revolutionize the traceability of authentic goods. PUFs have already started finding applications in field ranging from luxury goods^[^ ^229^ ^]^ to cell line traceability,^[^ ^265^ ^],^ and biocompatible PUFs could help tackle the key healthcare issues of food traceability and drug counterfeiting. Optical PUFs have emerged as a promising technology for anticounterfeiting and security applications, offering unique advantages over other (e.g., electronic) counterparts. They have witnessed fast and significant advancements, driven by the unique versatility of optically responsive materials, and by the diversity of compatible fabrication techniques. Optical PUF devices remain at a young developmental stage, and the perspective of introducing well‐established and/or well‐designed security inks, used in deterministic security devices, into novel unclonable devices has tremendous potential to push the field to next level. Indeed, while various optically active materials have been investigated as PUF components, some promising materials (e.g., photochromes) remain to be fully exploited in such devices. It is however worth noting that, although complex anticounterfeiting inks can be designed, their balance between cost and security increase is not necessarily favorable. The answer to high PUF encoding does not always lie in ink complexity, but potentially in the combination of multiple inks with orthogonal optical response. Combining optical readouts with other types of PUFs (e.g., resistance‐based) could also drastically improve the security level of the device.^[^ ^266^ ^]^ Moreover, the inherent randomness of PUFs can be introduced using a variety of (nano)technologies producing high levels of entropy, large information capacity, and potential resistance to machine learning attacks.

One of the most significant challenges that impact the effectiveness and practical application of optical PUF devices is reliability, and the choice of tag, matrix, and coating must be made accordingly. Optical PUFs must produce consistent outputs from repeated identical inputs to be reliable. However, variations within the same device can lead to different responses, known as intra‐chip variation. This can be caused by several factors, such as environmental disturbance, or temporal stability. Material photo‐ and physical stability must be high to design reliable optical PUFs. Additionally, environmental disturbances including temperature, humidity, and ambient light conditions can affect optical characteristics of PUF devices and lead to variations in output responses. For example, many optical PUFs require polymer matrixes to generate random patterns. Physical deformation of a polymer matrix caused by thermal expansion or moisture absorption could change its scatterer spacing and refractive index, leading to an increase in bit error rates. Optical PUFs utilizing random wrinkles or anisotropic materials can sometimes be sensitive to ambient light conditions since their optical characteristics depend on light transmission through these structures. The intensity and polarisation of incoming light can significantly affect how light interacts with the wrinkles, leading to variations in output responses. Background light can also interfere with fluorescence detection, degrade signal‐to‐noise ratio, and even introduce new noise to measurements. To decrease the influence of such environmental disturbances, several strategies can be considered. First, efforts can be made to develop new materials with both robust optical properties and minimal environment instability. Second, environmental control systems such as temperature‐controlled enclosures, moisture‐resistant coatings, and light‐tight enclosures can be involved to avoid inconsistent outputs. Furthermore, reference calibration systems and compensation algorithms can also mitigate environmental influences, enhancing the reliability of optical PUF devices. Temporal stability is also crucial for ensuring that the CRPs generated by the PUF remain reliable and reproducible. The aging of organic materials, photonic structures or polymer matrixes can lead to changes in their physical characteristics, such as structural integrity or optical clarity. Photo‐bleaching of fluorescent materials or fatigue of photochromic inks may also be important limitations to long‐term use. Considering that most valuable products could stay on the market for several years, it is necessary to investigate stability measurements over a long‐time scale and in real‐world conditions that could significantly impact device reliability.

The unclonable nature of PUFs must also be assessed and put into perspective. Nowadays, several technologies could potentially duplicate nanopatterns, which poses security concerns for optical PUFs. For example, electron beam lithography and focused ion beam can both generate nanostructures with sub‐10 nm resolution, which allows precise surface replication or unauthorized surface modification. High encoding can be achieved using large pattern areas or information‐dense PUF keys with multiple taggants. Introducing hidden features or dynamic elements can also reduce these security risks. For example, embedding codes or unique identifiers within the nanopatterns that are not visible without specific optical interrogation techniques. In this case, even if an attacker duplicates the pattern, they cannot easily derive valid authentication tokens. In this regard, “switchable” optical probes exhibiting reversible response to environmental factors (i.e., light irradiation, pH, etc.) are high potential inks for the design of PUF devices as they can be hidden within their structure and offer different readouts depending on the applied conditions. Besides dynamic materials, dynamic authentication mechanisms that change the CRPs over time can also reduce the effectiveness of cloned devices. For instance, periodically updating the parameters used in generating CRPs can enhance security. Other strategies, e.g., combining optical probes with significantly different lifetimes are also beneficial to increase the robustness of PUF devices. Based on a specific timeline, several nanopatterns can be collected from the same PUF, which makes the PUF challenging to simply duplicate by high‐resolution nanotechnologies.

Finally, for widespread use in the anticounterfeiting market, optical PUFs should be manufactured with technologies allowing large‐scale and relatively cheap production. In this sense, optical PUFs fabricated by techniques widely used in industry have significant potential for scalability and mass production. The scalability, time, and cost of the readout and authentication must also be in line with the end‐user. Advanced optical readout technologies requiring sophisticated equipment may therefore be more adapted to luxury designer goods than to the high‐throughput traceability of food products or medicine. Establishing benchmarking standards of encryption, performance, and reliability would also facilitate commercial applications. The NIST^[^ ^267^ ^]^ and the International Organization for Standardization (ISO)^[^ ^268^ ^]^ already provide guidelines and tests to evaluate the properties of PUFs (bit balance, runs of consecutive identical bits, entropy, etc.), and these standards should be more systematically considered in the literature. In summary, designing optical PUFs is a formidable engineering challenge that has potentially deep implications on both goods manufacturing and consumption. We have presented here a large selection of tags and processing technologies that will surely lead to the design of cutting‐edge unclonable anticounterfeiting devices. However, this abundance of options must be navigated carefully, by keeping in mind the pursuit of a balance between the design criteria presented here and the final purpose of the device represented by the product it will protect.

## Conflict of Interest

M.M.S. has invested in, consults for (or is on scientific advisory boards or boards of directors) and conducts sponsored research funded by companies related to the biomaterials field; has filed patent applications related to biomaterials; and has co‐founded companies in the biomaterials field. M.M.S. and J. Z. are listed as inventors on a pending patent application (2408586.2GB/PRV) describing an anticounterfeit device. The rest of the authors declare no conflict of interests.
